# Genomic, transcriptomic, and viral integration profiles associated with recurrent/metastatic progression in high‐risk human papillomavirus cervical carcinomas

**DOI:** 10.1002/cam4.3426

**Published:** 2020-10-05

**Authors:** Jing Jing Liu, Jung Yoon Ho, Jung Eum Lee, Soo Young Hur, Jinseon Yoo, Kyu Ryung Kim, Daeun Ryu, Tae Min Kim, Youn Jin Choi

**Affiliations:** ^1^ Department of Obstetrics and Gynecology Seoul St. Mary’s Hospital College of Medicine The Catholic University of Korea Seoul Republic of Korea; ^2^ Department of Obstetrics and Gynecology Yantai Affiliated Hospital of Bin Zhou Medical University College of Medicine Bin Zhou Medical University Yantai China; ^3^ Cancer Research Institute College of Medicine The Catholic University of Korea Seoul Republic of Korea; ^4^ Department of Medical Informatics College of Medicine The Catholic University of Korea Seoul Republic of Korea

**Keywords:** APOBEC, cervical cancer, human papillomavirus, immunoprofiling, metastasis, recurrence

## Abstract

Acquisition of recurrent/metastatic potential by a tumor cell defines a critical step in malignant progression. However, understanding of metastatic progression at the molecular level is scarce for cervical carcinomas (CES). In this study, we performed genomic, transcriptomic, and viral profiling of five pairs of primary (CES‐P) and matched recurrent/metastatic tumors (CES‐R/M) with high risk human papillomavirus. Whole exome sequencing revealed mutation features of CES‐R/M including elevated mutation burdens and prevalent copy number alterations compared to their matched CES‐P. A relative deficit of APOBEC‐related mutation signatures accompanying the transcriptional downregulation of *APOBEC3A* was observed for CES‐R/M. Mutations in genes encoding epigenetic regulators were commonly observed as CES‐R/M‐specific alterations. Immunoprofiling and gene set analysis revealed CES‐Ps were enriched with transcripts representing activated anticancer immunity such as interferon‐gamma pathway, while CES‐R/M exhibited upregulation of genes involved in epithelial‐mesenchymal transition and angiogenesis. Viral capture sequencing revealed that integration sites remained enriched in viral E1 protein domain during malignant progression. Moreover, we found transcriptional upregulation of *POSTN* and downregulation of *APOBEC3A* were associated with unfavorable clinical outcomes in CES. Comprehensive genomic and transcriptomic profiling of a rare cohort including CES‐R/M identified metastases‐specific features to advance the molecular understanding into CES metastatic progression with potential clinical implications.

## Graphical abstract

Mutations on genes encoding epigenetic regulators were observed as metastatic‐specific alterations. Immunoprofiling and gene set analysis revealed that primary cervical cancers were with activated anticancer immunity. Elevated POSTN and reduced APOBEC3A at transcription level correlated with unfavorable cervical cancer clinical outcomes.

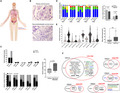

## 1 INTRODUCTION

Cervical cancer (CES) is the fourth most common cause of morbidity and mortality among women. Disease recurrence often accompanying locoregional or distant metastases is responsible for ~90% of cancer deaths.^1^ The majority of CES recurrence occurs locally (pelvis) and distant metastases occur in organs such as the lung (21%), bone (16%), para‐aortic lymph nodes (11%), abdominal cavity (8%), supraclavicular nodes (7%), liver (4%), and heart (<1%).^2^ Approximately 10% of patients at stages IB‐IIA experience relapse or metastasis following surgical treatment including radical hysterectomy. Median survival of patients with recurrent CES is only 7‐17 months and the majority (77%) of cases relapses within 3 years.^3^ The incidence of recurrence and metastasis of cervical cancer at stages IA, IB, IIA, IIB, III, and IVA within 10 years from the diagnosis is 3%, 16%, 31%, 26%, 39%, and 75% respectively. A substantial challenge for public health remains, given the relatively low 5‐year survival rate (<20%) for metastatic CES compared to that of localized cervical cancer (91.5% ).^4^

Carcinogenesis is an evolutionary process whereby cells or clones in the cancer mass continuously acquire genetic diversity in the form of somatic mutations, rendering them subject to clonal selection. Consequently, tumor subclones often display distinct genetic heterogeneity within a tumor mass. Heterogeneity can be sub‐classified into spatial and temporal genetic heterogeneity and is proposed to be the main cause of treatment failure, drug resistance, and tumor recurrence and metastasis.^5^, ^6^ Recently, advanced high‐throughput genomic analyses have enabled the genomic characterization of different types of human malignancies as reported in a large‐scale study from the Cancer Genome Atlas consortium.^7^ In addition to identifying clinically relevant genomic features, high resolution genomic profiling can be employed to study cancer evolution in that whole‐genome or whole‐exome sequencing‐derived mutations can serve as evolutionary markers to trace genomic evolution and spatial‐temporal heterogeneity. The comparison of multiple lesions obtained from a single patient including primary and metastatic lesions may also be valuable in identifying genomic features unique to metastatic lesions. Using multiregion biopsies from the same individual, investigators have evaluated spatial heterogeneity^8^, ^9^ as well as the genomic changes accompanying benign‐to‐malignant or primary‐to‐metastatic progression.^10^

The cancer transcriptome has also been reported to include a substantial number of transcripts originating from nontumor cells that can be exploited to investigate tumor microenvironments. For example, the relative abundance of tumor‐infiltrating immune cells and stromal cells can be estimated from bulk‐level transcriptome sequencing data.^11^ Moreover, such inferences can be further refined using previously acquired information such as the gene expression profiles of immune cells, as detailed in reports using the CIBERSORT algorithm.^12^ These approaches have become more important as immune checkpoint inhibitors have recently shown considerable clinical success^13^ Moreover, viral genome‐capture sequencing has been used to identify viral integration sites as genomic hallmarks of human papillomavirus (HPV)‐positive CES genomes.^14^

In the present study, we conducted integrative molecular profiling on multiple pairs of primary and recurrent/metastatic cervical cancers (CES‐P and CES‐R/M, respectively) from the same individuals, using three sequencing‐based methods: whole‐exome sequencing (WES), RNA sequencing (RNA‐seq), and high‐throughput viral integration detection (HIVID). We aimed to investigate: i) genomic and transcriptomic features of CES‐P and CES‐R/M and ii) CES‐R/M‐specific genomic and transcriptomic markers that may be associated with malignant progression of the disease.

## 2 MATERIALS AND METHODS

### 2.1 Patient samples

Paired normal, primary, and recurrent/metastatic tumor tissues were obtained from five cervical cancer patients (IB1‐IVB). All subjects underwent radical hysterectomy and adjuvant treatment after their first diagnosis and received either debulking surgery or biopsy upon recurrence or metastasis. HPV genotyping was performed on tumor samples; all patients were found to be infected with high risk HPV types (HPV16, 18, and 31). Ethical approval for the study was obtained from the relevant ethics committees. Formalin‐fixed paraffin‐embedded (FFPE) tissue blocks were micro‐dissected to obtain high‐purity (>70%) primary and recurrent/metastatic tumor tissues. A DNeasy Blood and Tissue Kit (Qiagen, Hilden, Germany) was used to extract the genomic DNA according to the manufacturer's recommendations.

### 2.2 Whole‐exome sequencing and DNA library preparation and qualification

A QIAamp DNA FFPE Tissue Kit (catalog number 56404; Qiagen) was used to extract the genomic DNA from tissue archives according to the manufacturer's recommendations. Exonic DNA capture was performed using an Agilent SureSelect Human All Exon 50 Mb kit (Agilent Technologies). A total of 500 ng DNA libraries were prepared and 101‐bp paired‐end sequencing reads were generated on an Illumina HiSeq2000 sequencer (Illumina). The Burrows‐Wheeler aligner (BWA) was used to align sequencing reads against a human reference genome (UCSC hg19, NCBI build 37.1).^15^ The Genome Analysis ToolKit (GATK) was used for local realignment and score recalibration of sequencing reads. Samtools and Picard were used to manage sequencing data.^16^ MultiQC v1.1 (http://multiqc.info/) was used for raw read‐ and alignment‐level quality control by aggregating QC metrics from FastQC (http://www.bioinformatics.babraham.ac.uk/projects/fastqc/) into an interactive report HTML and then was converted into PDF file (**Extended Data 1** and **2**). We used Mutect and Indelocator with the default settings to detect somatic mutations and indels by comparing tumor and matched normal sequencing reads (ie CES‐P and CES‐R/M sequencing reads were compared with their matched normal data, Table S1).^17^ Indels were filtered out with a stringent cut‐off (mutant allele frequency or MAF > 0.2). Mutations arising due to sequencing artifacts including FFPE‐related DNA fragmentation, were further filtered out using a software of FIREVAT (FInding REliable Variants without ArTifacts).^17^ The ANNOVAR package was applied to annotate the functional consequence of somatic mutations on the encoded amino acid residues.^18^ Tier1 mutations are those with strong evidence of their oncogenic or tumor suppressive roles as presented in the Cancer Gene Census (CGC, https://cancer.sanger.ac.uk/census). We also classified somatic mutations into three regional categories, with common mutations including those observed in both CES‐P and CES‐R/M in given cases and CES‐P‐ and CES‐R/M‐private mutations representing those observed only in the corresponding lesions.

### 2.3 Mutational signature analysis

A total of 65 mutation signatures as normalized frequencies of 96 trinucleotide contexts representing six substitution subtypes (ie C>A, C>G, C>T, T>A, T>C, and T>G) along with bases immediately 5ʹ and 3 ʹ, were obtained from COSMIC database.^19^ (https://cancer.sanger.ac.uk/cosmic/signatures/SBS/; mutation signatures version 3). The mutation signature levels or weights from a mutation profile (ie the frequency of mutations in the context of 96 trinucleotides) were estimated using the deconstructSigs R extension.^20^ We performed mutation signature analysis for three mutation categories, ie, the common and CES‐P‐/CES‐R/M‐private mutations for each patient. Among the mutation signatures, we selected 16 signatures with known causal relationships, ie, SBS1/2/3/4/6/7a/7b/7d/10a/10b/13/14/15/20/26/44, and others. The mutation signature weights were combined when possible (eg SBS 2/13 associated with APOBEC activity and SBS6/14/15/20/26/44 associated with a deficiency of DNA mismatch repair, respectively).

### 2.4 Clonal mutational structures

The number of mutation clones of given cases were inferred by SciClone (version 1.1.0)^21^ (Extended Data Figure 1). SciClone applies a Bayesian clustering to the joint mutation profiles of paired samples such as longitudinal or multiregion biopsies (spatially or temporally separate) samples to infer the sub‐clonal mutation architecture and their evolutionary dynamics of a given cancer genome.

**FIGURE 1:**
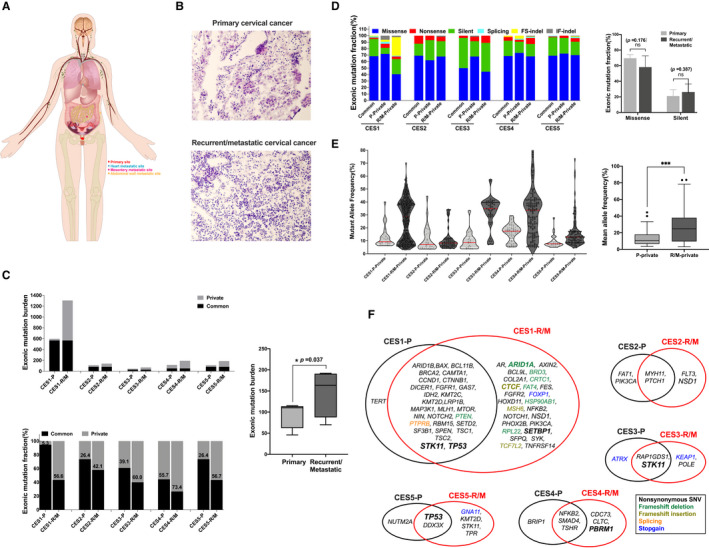
Mutation abundance and features of primary and matched recurrent/metastatic cervical cancers. A, Schematic diagram of cervical cancer with different metastatic sites recruited in this study. B, Microscopic features of primary cervical cancer(top), and metastatic tumor on mesentery(bottom) by hematoxylin and eosin(H&E) staining. C, Abundance of exonic mutations(upper panel) are shown along the *y*‐axis across five cases with each primary (P) and recurrent/metastatic (R/M) sample. Mutation fractions(lower panel) are further classified into common and primary mutations (black and grey, respectively). **P‐value < .01 D, The relative abundance of six categories representing the functional consequences of somatic mutations are shown and the relative abundance of missense and silent mutations are compared between CES‐P and CES‐R/M tumors. E, The mutant allele frequencies (MAFs) are shown for CES‐P and CES‐R/M genomes with comparison of matched genomes. F, Cancer Census Genes (Tier‐1) are listed for three regional categories of common, P‐, and M‐specific driver mutations for five cases

### 2.5 Copy number profiling

For copy number profiling, we used the Excavator algorithm based on read depth differences between the tumor and matched normal sequencing data.^22^ To define amplifications and deletions, we set thresholds for the log2 ratios (eg, amplification and deletion for >0.2 and < –0.2 log2 ratio, respectively). According to the overlap between CES‐P and CES‐RM genomes, individual segments were classified as common or private to CES‐P or CES‐R/M. The visualization of copy number profiles was prepared using the IGV browser.

### 2.6 RNA sequencing

RNA extraction was performed with the FFPE RNeasy kit (Qiagen, catalog 73504) according to the manufacturer's instructions under sterile RNase/DNase‐free conditions. RNA concentration was determined with the NanoDrop (ND‐1000) spectrophotometer (Thermo Fisher. Scientific, Inc). Transcriptome analysis of CES‐P and CES‐R/M was performed on an Illumina HiSeq2500 system (Illumina). A TruSeq RNA Access Library Prep Kit (Illumina) with 50 ng RNA was used to prepare cDNA libraries according to the manufacturer's instructions. Sequencing read quality was assessed for pre‐mapped sequencing reads of R1 and R2 FASTQ files as well as aligned reads in BAM files using FASTQC (http://www.bioinformatics.bbsrc.ac.uk/projects/fastqc). Low quality reads were filtered out and the remaining reads were hard trimmed using Trimmomatic. The cleaned‐up reads were aligned using the Tophat2 splice‐aware aligner.^23^ Pre/post‐mapped sequencing reads were visualized for QC metris using MultiQC v1.1 (http://multiqc.info/) (**Extended Data 3** and **4**). FPKM (fragments per kilobase million) values were obtained from CuffLinks^24^ and raw count values were calculated with the featureCounts tool using the Galaxy web‐based platform (https://usegalaxy.org/). For transcripts, we used annotations derived from hg19 annotations (UCSC, TCGA.hg19.June2011.gaf). *P*‐values were obtained by a paired *t* test between the primary and matched recurrent/metastatic tumor groups and fold changes were calculated. Differentially expressed genes (DEGs) were identified as those with log2 |(fold change)|> 1 and *P*‐values < .05 using DESeq2.^25^

### 2.7 Immunoprofiling

The CIBERSORT deconvolution algorithm^26^ was applied to the CES‐P and CES‐R/M RNA‐seq data to estimate the relative abundance of 22 immune cell subtypes. CIBERSORT algorithm is based on support vector regression and known to provide reliable estimates on the relative abundance of immune cells.

### 2.8 Gene set enrichment analysis (GSEA)

To identify the functionally coordinated gene expression changes between CES‐P and CES‐R/M, we applied GSEA (version 2.0, http://software.broadinstitute.org/gsea) using three types of functional gene sets as available in the MSigDB databas (http://software.broadinstitute.org/gsea/msigdb/index.jsp). The gene set categories used in the analysis were H (Hallmark), C2cp (curated pathways), and C5 (Gene Ontology) gene sets. The enrichment score (ES) or normalized ES along with permutation‐based p‐values were calculated for individual gene sets with multiple test adjustments. A conventional threshold *P*‐value < .05 and false discovery rate (FDR) < 0.25 were considered significant.

### 2.9 Human papillomavirus integration analysis (HIVID)

Genomic DNA was extracted using a QIAamp DNA FFPE Tissue Kit (catalog number 56404; Qiagen) according to the manufacturer's protocol. gDNA fragmentation, library preparation and sequencing were performed as described previously.^27^ HPV typing was done for five pairs of CES‐P and CES‐R/M cases (Table 1) using HPV probes^28^ designed by MyGenostics (MyGenostics, Baltimore, MD, USA). Structural variation detection algorithms of SVDetect^29^ and CREST^30^ were used to identify spanning reads or soft‐clipped reads supporting the HPV integration sites.^27^ The paired end sequencing read, with one end uniquely aligned to the human genome (hg19 with chrY excluded) and another to the HPV genome, is defined as a discordant read pair.^31^ A potential HPV integration locus requires one or more discordant read pairs^32^ and at least one split read.^33^ Based on the experimental threshold from MyGenostics GenCap Technology, the local‐assembled sequence of more than three. soft‐clipped reads consistent with the integration site reads, is considered to be a high‐confidence site.HPV integration sites supported by either of the algorithms (SVDetect or CREST only) are low‐confidence sites.

**TABLE 1:** Clinico‐pathologic information of the five cervical patients

Patient ID	Age	Diagnosis	FIGO stage	Histologic differentiation	Metastasis site	HPV types	HPV typing by HIVID	Concurrent cancer
CES1	52	SCC	IVA	Grade 3	Mesentery	16	N/A	Colon cancer
CES2	44	SCC	IIB	Grade 2	Abdominal wall	18	18	—
CES3	58	ASC	IB1	Grade 3	Abdominal wall	16	16	Breast cancer
CES4	34	SCC	IIA2	Grade 3	Heart	16	16	—
CES5	49	SCC	IB1	Grade 3	Abdominal wall	31	31	—

Abbreviations: ASC, Adenosquamous cell carcionma; N/A, Not available; SCC, Squamous cell carcinoma.

### 2.10 Statistics analysis

Differential mutation abundance and gene expression between the primary and matched recurrent/metastatic tumor groups were analyzed using paired Student's *t* test. Overall survival (OS) and disease‐free survival (DFS) were evaluated using the Kaplan‐Meier method and the significance level was estimated using the log‐rank test. Statistical analyses were performed with GraphPad Prism version 8.0 software (GraphPad Software, Inc). Error bars are shown, all data are represented by median ± standard deviation (SD). Significance was defined as a *P*‐value less than .05. All statistical tests are two‐tailed, unless otherwise specified.

## 3 RESULTS

### 3.1 Exonic mutations of CES‐P and CES‐R/M

We noted a clear bimodal distribution of GC contents for the forward and the reverse reads across the cases examined. No other discernible systematic problems were noted for the WES data (Extended Data 1 and 2). The median age of the five patients was 52 years (with a range of 34‐58 years) (Table 1). Recurrent/metastatic CES occurred in the abdominal wall (3/5 patients), mesentery (1 patient), and heart (1 patient) (Table 1,Figure 1A). Representative images for primary and matched recurrent/metastatic tumor from CES1 mesentery site disclosed that cell morphology was more poorly differentiated in a large heterotypical change after recurrence (Figure 1B). We first identified two types of somatic mutations—single nucleotide substitutions (SNV or point mutations) and short insertions/deletions (indels)—from the WES data (hereafter, collectively referred to as somatic mutations). We compared the number of exonic mutations or mutation burdens between five pairs of CES‐P and CES‐R/M (Figure 1C). A total of 979 and 1894 somatic mutations identified in CES‐P and CES‐R/M, respectively, are available in Table S2. One of five cases (CES1) exhibited hypermutated genomes, with a high level of mutation burdens for CES1‐P and CES1‐R/M harboring 598 and 1305 somatic mutations respectively (Figure 1C). The mutation burdens of four recurrent/metastatic tumor samples (CES2‐R/M–CES5‐R/M, median 103.5; 70‐192) were statistically significantly higher than those of matched primary tissues (median of 110 mutations, 46‐115) (Figure 1C, **P‐value = .037; paired *t* test). We observed that 55.1% (26.4‐94.6%) of all SNVs were shared between CES‐P and matched CES‐R/M, representing common ancestral mutations (common mutations). For lesion‐specific mutations, CES‐P‐ or CES‐R/M‐specific mutations (as primary‐ or metastasis‐private mutations) comprised 30.6% (5.4‐55.7%) or 59.2% (49.0‐73.4%) of mutations in given genomes respectively (Figure 1C, Table S2). The dominance of CES‐R/M‐specific mutations over CES‐P‐specific mutations suggests that CES‐R/M genomes acquired more mutations than the CES‐P genomes after metastatic dissemination; this also explains the higher mutation burdens of CES‐R/M compared to those of CES‐P.

For three regional mutation categories (common, P‐, and R/M‐private mutations), the consequences of mutations on encoded amino acids and mutant allele frequencies (MAFs) were further investigated (Figure 1D,E respectively). For CES1‐R/M, frameshifting indels were prevalent compared to other genomes. The predominance of mutations and indels in CES1 suggests that this case may carry DNA repair deficiency, elevating the accumulation of both SNVs and indels. No significant differences between the ratio of missense and silent mutations were observed (Figure 1D). For MAFs, CES‐R/M genomes showed significantly elevated levels of MAFs (12.88% ± 7.69%) compared to matched CES‐P (25.13% ± 16.08%) (Figure 1E, ***P‐value < .001). Lower MAFs in CES‐P compared to CES‐R/M suggested that the mutational subclonal architecture of primary tumors was more heterogeneous than that of metastatic tumors that may have undergone clone‐level selection during metastatic dissemination. The initial seeding of metastatic clones originating from a single cell or oligoclonal cells from primary tumors must be clonal or oligoclonal, representing likely events of clonal selection associated with metastasis. In addition, the growth of metastatic tumors in lineage‐unrelated microenvironments may allow for the acquisition of new subclonal mutations leading to increased mutation burdens of CES‐R/M respectively. The newly acquired mutations—most of which remained subclonal without becoming fixed in the population—may constitute the subclonal mutations present in CES‐R/M. In addition, the evolutionary dynamics associated with the metastatic progression was monitored by estimating the number of mutation clones between matched primary and metastatic genomes. The number of mutation clones were inferred using SciClone algorithm, which indicates the extent of heterogeneity of given cancer genomes. For two cases (CES1 and CES4), CES‐R/M genomes showed more number of mutation subclones compared to CES‐P genomes (eg, 2 and 4 mutation clones in primary and metastasis of CES1; 2 and 5 mutation clones in primary and metastasis of CES4, respectively, Extended Data Figure 1). For these tumors, metastatic progression accompanies the emergence of new sub‐clones that might drive tumor growth with interactions between different sub‐clones.^34^ Moreover, for other tumors, the clone numbers were largely concordant between primary and metastasis (eg, 2/2 for CES1, 2/2 for CES2 and 2/5 for CES4) suggesting the evolutionary bottleneck during metastatic progression is not supported for these cases.

The number of cancer‐related genes occurring in both primary and metastatic cervical cancer was also variable among patients (Figure 1F). Among the mutations involving cancer‐related genes, frequent somatic mutation events were observed in *STK11* and *TP53*, as common mutations shared by CES‐P and CES‐R/M in three cases (Figure 1F), with similar frequencies of alterations as previously reported for cervical cancers.^35^ For lesion‐specific mutations, missense mutations on *NSD1* encoding histone H3 lysine 36 methyltransferase were observed as CES‐R/M‐specific mutations in two cases (CES1 and CES2). *NSD1* mutations are frequently observed in head‐and‐neck cancers^36^ and are likely loss‐of‐function mutations leading to genome‐wide hypomethylation.^37^

In addition, we observed a number of CES‐R/M‐specific mutations in epigenetic regulators such as *ARID1A*, *ARID1B*, *CTCF*, *KMT2C*, *KMT2D*, *PBRM1*, and *SETBP1*, suggesting that metastatic progression of CES may be associated with loss of epigenetic regulation. Among these epigenetic modifiers, ARID1A mutations may serve as therapeutic targets using synthetic lethality. For example, the inhibition of EZH2—the synthetic lethal partner of ARID1A—caused tumor regression in *ARID1A*‐mutated ovarian cancer cells^38^ and associated with hepatocellular carcinoma metastases.^39^

### 3.2 Mutation signatures

**FIGURE 2:**
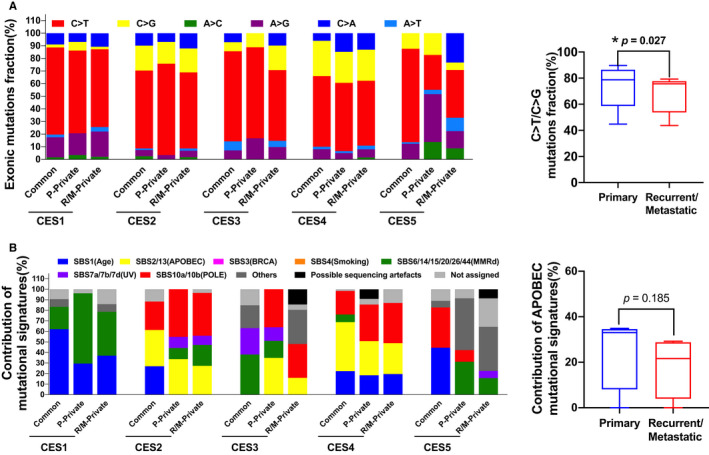
Mutation signatures between primary and matched recurrent/metastatic genomes. A, The six mutation spectra are shown for three regional categories. The frequencies of C>T/C>G substitutions are compared between CES‐P and CES‐R/M. B, Relative proportion of eight representative mutation signatures are shown for three regional categories. For the top 16 most frequent mutation signatures, those with similar causal relationships are combined (eg, SBS2 and SBS13) and the remaining, less‐frequent mutation signatures are collected as others. For APOBEC‐related mutation signatures (SBS2 and SBS13), the relative contributions are compared between CES‐P and CES‐R/M

Based on mutation spectra, C>T and C>G substitutions were the most common types of mutations in cervical cancers (in common, P‐private, and R/M‐private) (Figure 2A). We further performed further mutation signature analysis to deconvolute the mutation profiles of individual cancer genomes into a set of relative weights or contributions of distinct signatures. We used 65 SBS (single base substitution) mutation signatures as available in the database (COSMIC ver. 3 mutation signatures).^12^ Seven major signatures are shown for their relative contribution (Figure 2B). Except for CES1, SBS2‐SBS13 representing APOBEC overactivity yielding both C>T transitions and C>G transversions in the context of dipyrimidines were prevalent across the cases as a previous paper reported.^21^ A significant reduction in the levels of C>T transitions and C>G transversions (Figure 2A, *P*‐value = .027) associated with a relative deficit of APOBEC‐associated signature (SBS2‐SBS13) was found in three CES‐R/Ms, compared to the matched CES‐Ps (Figure 2B). It has been reported that *APOBEC* mRNA levels can serve as a proxy to APOBEC activity level as well as the abundance of the associated mutations.^24^, ^25^ Our transcriptomic analysis also demonstrated that the *APOBEC3A* mRNA levels—but not other APOBEC family members—were significantly higher in CES‐P than in CES‐R/M (*P*‐value = .047; Table S3, Figure S1), suggesting that decreased *APOBEC3A* expression in CES‐R/M may be responsible for the reduced levels of SBS2‐SBS13. In the case of CES1, Sig6—representing mutations associated with a deficiency of DNA mismatch repair (MMRd)—was dominant along with age‐related mutation signatures, suggesting that MMRd may be responsible for hypermutations along with a predominance of indels in CES1.

### 3.3 Transcriptomic analysis

**FIGURE 3:**
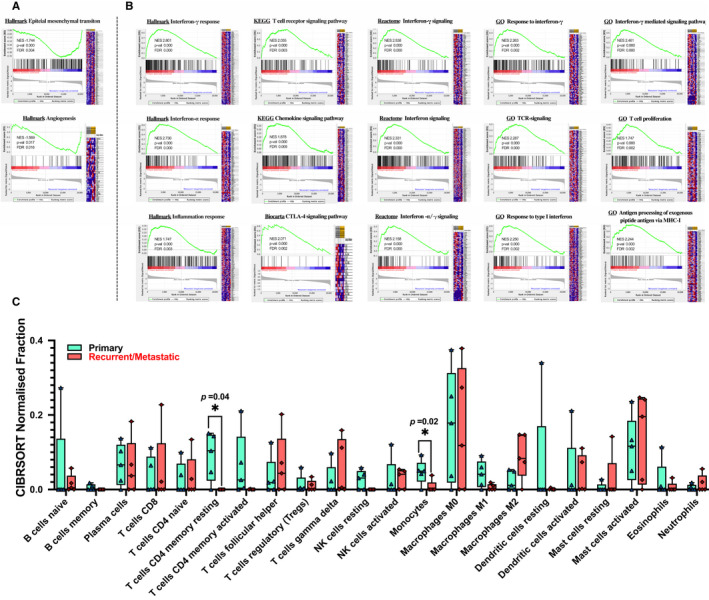
Transcriptome‐based functional gene set and immunological analysis of CES‐P and CES‐R/M. A, Enrichment plots of epithelial‐to‐mesenchymal transition and angiogenesis hallmark genetic sets whose gene members are relatively upregulated in CES‐R/M compared to CES‐P are shown. B, Immune‐related gene and inflammation‐related sets whose gene members are relatively upregulated in CES‐P compared to CES‐R/M are shown. Gene sets are as available in MSigDB, Hallmark (H), KEGG, Biocarta, Reactome (all in c2), and Gene Ontology (GO; c5 categories). C, Immune cell abundance (*x*‐axis) estimated by the CIBERSORT algorithm for 22 immune cell subsets are shown. *P‐value < .05

The base quality control was performed for transcriptomic sequencing data (Extended Data 3 and 4). To identify functional changes associated with the metastatic progression of CES, we conducted GSEA analysis using functional gene sets available in the MSigDB database (http://software.broadinstitute.org/gsea/msigdb/). For hallmark gene sets, top significantly enriched gene sets for genes upregulated in CES‐R/M compared to CES‐P are those representing epithelial‐to‐mesenchymal transitions (EMT) and angiogenesis (Figure 3A; FDR < 0.01). In addition, immune‐ and inflammation‐related gene sets were upregulated in CES‐P compared to CES‐R/M (eg T cell receptor signaling pathway and chemokine signaling, CTLA‐4 signaling pathway and interferon signaling) (Figure 3B and Tables S4 and S5). To further estimate the level of individual tumor‐infiltrating immune cells in CES, we performed CIBERSORT. As shown in Figure 3C, the relative abundance of immune cells is variable across samples. We observed that resting memory CD4 T cells as well as monocytes were enriched in CES‐P in comparison with CES‐R/M (Figure 3C, *P*‐value < .01).

### 3.4 HPV integration in the human genome

**FIGURE 4:**
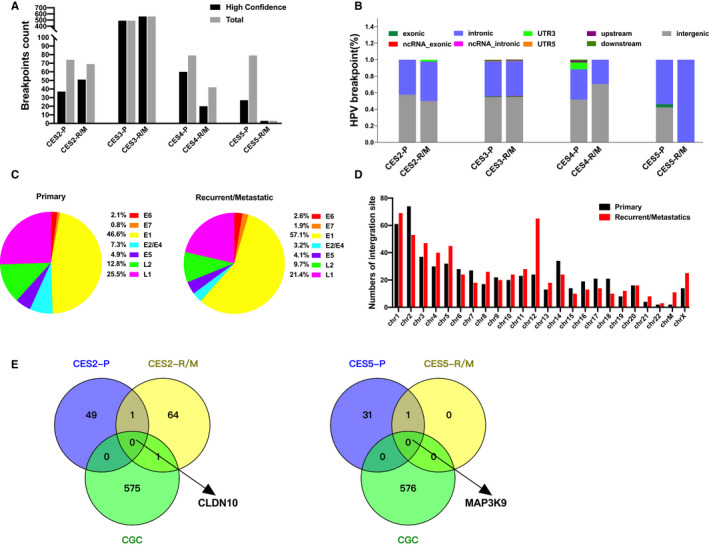
HPV integration on CES genomes. A, The abundance of HPV integration is shown in two measures, total and high‐confidence integrations. B, Ratio of HPV breakpoints in variants classified according to genomic region with untranslated region promoter, enhancer, exon, intron, and intergenic region included in primary and recurrent/metastatic types. C, Pie chart of breakpoints occurring in the annotated regions of the HPV genome. D, Genome‐wide distribution of HPV integration breakpoints across the human genome. E, Venn diagrams show the number of HPV integration sites common or private to the CES‐P and CES‐R/M. Genes corresponding to the HPV integration sites are shown with gene symbols

It has been reported that the persistent presence of HPV DNA in distant metastases of cervical carcinoma could play a crucial role in the acquisition and maintenance of malignancy‐related features.^40^ New cases of ovarian neoplasia confirmed the cervical origin of the ovarian metastasis which was supported by the same HPV genotypes and their integration sites in the paired cervical and ovarian tumors.^41^ To investigate whether HPV integration contributes to cervical cancer recurrence and metastasis, we used the HIVID technique to identify the integration sites of HPV for four pairs of CES‐P and CES‐R/M (CES2‐CES4). A total of 633 HPV integration breakpoints were detected in four CES‐P and 614 integration events in CES‐R/M. An average of 214 and 234 high confidence breakpoint counts were detected in CES‐P and CES‐R/M respectively (Figure 4A). HPV integration breakpoints were highly enriched in the intergenic and intronic regions in all samples, with a comparable integration frequency in each region between CES‐P and CES‐R/M (Figure 4B). The observed breakpoints were scattered throughout the entire HPV genome, but were enriched in E1 in both CES‐P and CES‐R/M genomes (Figure 4C)—consistent with previous reports.^42^ Persistent HPV infection that leads to carcinoma is characterized by overexpression of E6/E7 and loss of E2 functions to induce cellular proliferation and viral immortalization.^42^, ^43^ We also detected a higher number of breakpoints in E6, E7, and E1 and a lower number of breakpoints in E2/E4, and L1/L2 in CES‐R/M compared to those in CES‐P (Figure 4C). Regarding viral integration sites in host genomes, 10.5% (68/673) of integration sites discovered in CSE‐R/M genomes were localized on chromosome 12, compared to 3.7% (27/722) in CSE‐P (Figure 4D and Table S6). HPV integration in intronic regions of *CLDN10* and *MAP3K9* with roles in the tight junction and MAPK signaling pathways, respectively, were also observed in both CES‐P and CES‐R/M samples (CES1 and CES5, respectively) (Figure 4E).

### 3.5 Copy number profiling

**FIGURE 5:**
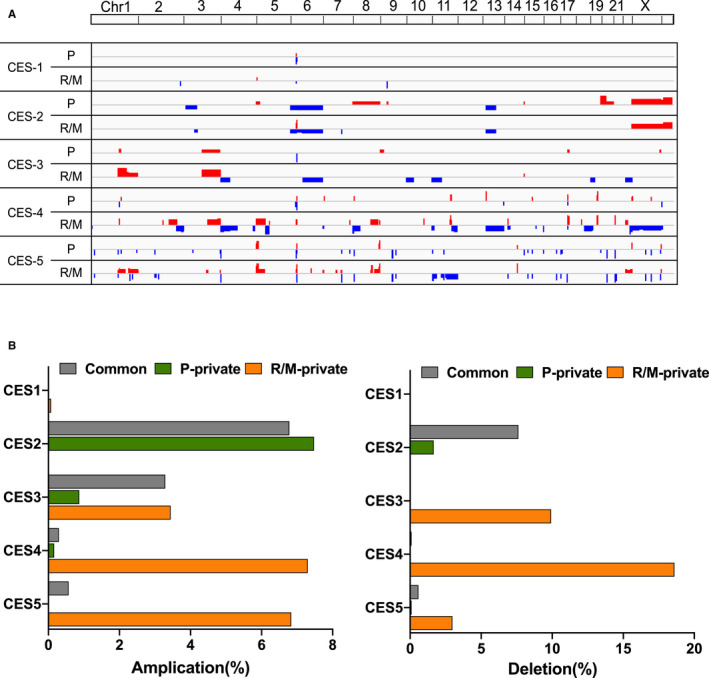
Copy number profiling. A, Genome‐wide heatmap representing the somatic copy number alterations (sCNAs) in the discovery cohort is shown. Red and blue represent chromosomal amplifications and deletions, respectively. B, Chromosomal segments are further distinguished into three regional categories of common, P‐, and R/M‐private SCNAs. The genomic percentages of three categories of SCNAs are shown for five cases separately for chromosomal amplifications and deletions (left and right, respectively)

It has been proposed that well‐recognized SCNAs (somatic copy number alterations) in CES, including 3q gains, represent markers of stepwise disease progression and adverse clinical outcomes.^44^ Genome‐wide SCNA profiles of five CES pairs are shown in Figure 5A. A lack of SCNA was observed for CES1, consistent with a relative deficit of SCNA for hypermutated genomes such as MSI‐H genomes. The extent of concordance of SCNAs between CES‐P and CES‐R/M were variable across the cases. For example, CES2 harbors three SCNAs (loss of 6p/q and 13p along with gain of Xp/q) as common SCNAs that emerged before dissemination of metastatic tumors. For CES2, loss of 3p along with gain of 8p/q and 20p/q were CES‐P specific and not observed in CES‐R/M, suggesting that the CES‐R/M cases did not acquire additional SCNAs after dissemination compared to parental, primary genomes. To the contrary, for CES3, a single SCNA (3q gain) was common while a majority of lesion‐private SCNAs were observed as CES‐R/M specific (e.g, gain of 1q and loss of 4p, 6q, 10p, 11p, 19p, and 22q). The dominance of CES‐R/M specific SCNAs was also observed for CES4 and CES5 (Figure 5B). Thus, the concordance level between CES‐P and CES‐R/M was variable, but the dominance of CES‐R/M‐specific SCNAs was often observed, suggesting that aneuploidy level is elevated in CES metastatic genomes, probably with genomic instability. The number of genes and cancer‐related genes belonging to common, CES‐P‐private, and CES‐R/M‐private SCNAs in each case are available in Table S7.

### 3.6 Predictive biomarkers based on gene expression profiles

**FIGURE 6:**
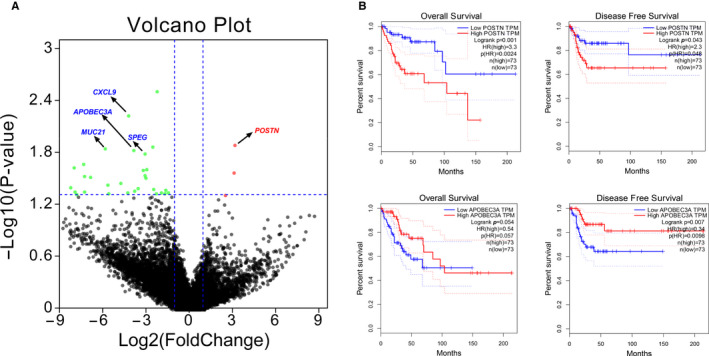
Relationship of overall survival rate and disease‐free survival rate with candidate potential biomarkers. A, Differentially expressed genes based on the DESeq2 algorithm driven by volcano plot; red dots represent upregulated genes, green dots represent downregulated genes with a threshold log2 |(fold change)|> 1 and *P*‐value < .05. The decreased expression levels of *APOBEC3A*, *CXCL9*, *MUC21*, and *SPEG* and increased *POSTN* expression level marked the top genes when the threshold cutoff was set at log2 |(fold change)|> 3 and *P*‐value < .02. B, Overall survival and disease‐free survival curves of potential DEGs were assessed by Gene Expression Profiling Interactive Analysis (GEPIA) based on primary cervical cancer profiles from The Cancer Genome Atlas database (TCGA); high/low gene expression groups were cutoff at the quartile, *P*‐value < .05

To identify expression‐based biomarkers with potential clinical implications, we first identified 36 differentially expressed genes (DEGs) using the DESeq2 algorithm (ie,> 2‐fold changes and *P*‐values < .05, Table S3) between CES‐P and CES‐R/M. DEGs include *APOBEC3A*, *CXCL9*, *MUC21*, and *SPEG*, which were significantly downregulated in CES‐R/M, while the expression of *POSTN* was significantly upregulated in CES‐R/M compared to CES‐P (Figure 6A). We then examined whether the selected DEGs have prognostic implications using GEPIA online tools (http://gepia.cancer‐pku.cn/). For clinical evaluation, we used the expression and patient survival information for cervical cancer available from the TCGA consortium. The analysis revealed that the transcriptional downregulation of *APOBEC3A* expression was associated with poor DFS and OS in human cervical cancers (*P*‐value = .007 and 0.054, log‐rank test). Transcriptional upregulation of *POSTN* expression was significantly associated with poor OS rate and DFS rate (*P*‐value = .001 and .043, respectively, log‐rank test, Figure 6B), indicating that POSTN may serve as a prognostic biomarker for metastatic cervical cancer.

## 4 DISCUSSION

In this study, we report a comprehensive genomic and transcriptomic landscape of five cervical cancer patients by examining the matched pairs of primary and recurrent/metastatic specimens. Despite the availability of a large public database for primary cervical cancer, genomic datasets for metastatic cervical cancer have been lacking, given the inherent difficulties of performing biopsies or secondary debulking surgeries when recurrence occurs. To the best of our knowledge, this is the first study to characterize somatic alterations, copy number variations, gene expression changes, immune profiles, and the genetic variability of HPV types in primary cervical cancers and their matching recurrent/metastatic cancers. The metastasis‐specific genomic and transcriptomic features may uncover molecular insights into the mechanisms likely driving the mutational, transcriptomic, and viral landscape of cervical cancer recurrence.

APOBEC cytidine deaminases plays a crucial role in innate immune response and viral restriction by removing the amino group from a cytosine, converting it to uracil. Prolonged APOBEC activation during HPV infection can induce somatic mutations in the host genomes, thereby contributing to the initiation and progression of the disease. However, it remains unclear whether APOBEC enzymes cause a significant fraction of the driver mutations or genome rearrangements in cervical cancer recurrence/metastases. Recent reports revealed that the overactivity of APOBEC was associated with the generation of late‐occurring subclonal mutations in the evolution of primary tumors, serving as source for intratumoral heterogeneity.^45^ In our study, APOBEC mutagenesis was the most prominent signature in the majority of samples, consistent with a recent report.^46^ Moreover, a substantial depletion of C>T transitions and C>G transversions representing APOBEC mutagenesis (SBS 2/13) accompanying a decreased expression of APOBEC3 genes, was observed in metastatic samples when compared to primary samples. Our results are supported by a recent study that reported APOBEC signatures in stock cervical cancer cell lines but no discernible continuing activity in their daughter lineages.^47^ In addition, the study reported that tumors with enriched interferon signaling pathways (eg, IFN‐γ) were associated with high APOBEC3 expression and that increased expression of *STAT1*, a downstream effector of IFN‐γ, was accompanied by an increased expression of *APOBEC3A*; these results are consistent with our data (Figures 2A and 3B).

In our study, one patient (CES1) lacked the APOBEC signature in both primary and recurrent/metastatic tumors, instead showing the prevalence of mutation signatures associated with MMRd. This patient also showed concurrent colon cancer with an *MLH1* missense mutation, implying Lynch syndrome.^48^ We previously showed that 2.3% of CES cases show MSI‐H genotypes,^49^ the proportion of which is relatively low compared to colorectal and uterine cancers. Our results suggest that concurrent cancers with clinical implications of Lynch syndrome‐including CES—should be considered for the presence of hypermutation.

In cervical cancer, the inflammation induced by HPV leads to a complex immune milieu with various types of tumor‐infiltrating immune cells that play an important role in tumorigenesis. Recently, Chen et al revealed that immune cell composition can be substantially different between tissues with inflammation and cancer. For example, resting memory CD4^+^ T cells, activated mast cells, monocytes, and M1 macrophages are more abundant in inflammatory stages, while CD8^+^ T cells, follicular helper T cells, and M0 macrophages are more abundant in cancer tissues.^50^ Our CIBERSORT analysis showed that monocyte fractions were relatively lower in metastatic samples along with a deficit of immune‐related and inflammation‐related pathway activation. This finding is in accordance with previous research reporting that classical monocytes are recruited at higher rates by inflamed tissues and are able to attract other immune cells by secreting cytokines and antimicrobial factors.^51^, ^52^ Lack of memory CD4+ T cells in metastatic samples is associated with impaired IFN‐related signaling, given their close correlation with IFN‐γ production and inflammation activity. Moreover, our GSEA pathway enrichment data reveals that recurrent/metastatic cervical cancer samples exhibit impaired inflammation, chemokines, immune activity, interferon, T‐cell signaling, and antigen processing signaling pathway, all of which are regarded as characteristics of “cold/non‐inflamed” tumors with poor response to immunotherapy.^53^

Viral integration into intergenic and chromosome fragile sites frequently occurs in cervical cancers, resulting in the impairment of E2 and increased expression of E6 and E7.^43^, ^54^ Relatively enriched breakpoints in E6 and E7 and the disruption in E1 genes of CES‐M were observed in comparison with CES‐P. Although no recurrent high confidence HPV integration sites were observed among the metastatic samples, some of the singleton events were observed in genes with potential tumorigenic effects. For instance, in CES2, HPV integration was observed in *CLDN10*,^55^ the loss‐of‐function of which may lead to the loss of cellular adherens junctions in EMT pathways,^56^ contributing to tumor metastasis.^55^ HPV integration into the *MAP3K9* gene was also observed in CES5.

Tumor mutational burden (TMB) has been proposed as a biomarker to predict the therapeutic response to immunotherapies.^57^, ^58^ In our study, significantly higher TMB was found in CES‐R/M compared with CES‐P. Higher mutation burdens in metastatic tumors compared to primary tumors were also reported in a large‐scale genomic study on metastatic breast cancer.^59^ Higher MAFs of mutations in CES‐R/M genomes also suggest the presence of clonal selection events that are relatively unique to CES‐R/M genomes. We did not observe any significant change in nonsynonymous mutations, which was reported to be an improved objective response rate factor between CES‐P and CES‐R/M according to our study. Common mutations found to be altered in both the primary lesions and their metastases represent early cancer drivers, while the lesion‐specific or private mutations are those acquired after metastatic dissemination. In addition, metastatic‐specific mutations may serve as potential markers involved in metastatic progression. Wingo et al reported 20% of cervical cancers harbor somatically‐acquired *STK11*mutations^35^ and can suppress the tumor immune surveillance response by directly impacting NF‐κB activity and other pathways in lung adenocarcinoma.^60^ *STK11* mutation confers resistance to immune checkpoint inhibitors.^61^ In our study, *STK11* harbored recurrent mutations for two patients along with recurrent chromosomal losses in two metastatic samples, suggesting that *STK11* alterations confer the ability of cervical cancers to metastasize.

Using transcriptome analysis, we found two potential predictive biomarkers (*APOBE3A* and *POSTN*) for recurrent/ metastatic cervical cancer. We determined that the transcriptional downregulation of *APOBEC3A* dictated a poor clinical outcome including abbreviated disease‐free survival. The association of APOBEC expression and activity with disease progression remains contradictory, especially with regard to HPV infection. For example, higher expression levels of *APOBEC3A* can be induced by HPV16 infection in oropharyngeal cancer,^62^ while APOBEC3A protein levels were downregulated during invasive progression in HPV‐negative but not HPV‐positive penile squamous cell carcinomas, suggesting that HPV‐associated APOBEC3A activity is variable and context‐dependent.^63^ Given that decreased *APOBEC3A* levels were observed as CES‐R/M group‐specific features and as unfavorable clinical outcome‐predicting features, it is assumed the APOBEC activity is repressed in metastatic tumors and may predict the disease progression and metastasis in CES with HPV infection. The second potential predictive biomarker we identified was *POSTN*. It is thought to be a crucial player in cancer development and metastasis,^64^ as well as chemotherapy resistance^65^ across different tumor types. High *POSTN* levels in tumors are associated with aggressive tumor behavior as well as advanced stage and/or poor prognosis in colorectal cancer.^66^ All of these reports support our finding that upregulated expression of *POSTN* is associated with poor overall survival and disease‐free survival rates in TCGA primary cervical cancer, identifying upregulated *POSTN* level as a novel potential predictive biomarker for cervical cancer recurrence/metastasis as studies in many other cancers.^66^, ^67^, ^68^ An extended cohort will be required to validate the potential biomarkers that we identified in our analysis.

This study has several limitations. First, our study had a small sample size precluding definite conclusions which normally require a larger sample size to ensure a representative distribution of the population and to be considered representative of groups of people by statistical tests. Second, the use of FFPE tissue archives instead of fresh‐frozen (FF) tissues may be associated with known artifacts with impacts on the sequencing and the fidelity of expression profiles as previously proposed with limited quantity of DNA from FFPE samples.^69^, ^70^, ^71^ Lastly, the validation of targeted RNA‐seq by reverse transcriptase polymerase chain reaction (RT‐PCR) and HPV integration sites by fluorescent in situ hybridization (FISH) or PCR should be considered in the further study.

In summary, due to difficulties in the acquisition of matched tissue biopsies from cervical cancer patients, hopefully, our results can be of help to pathologists and clinicians alike to identify both genome‐ and transcriptome‐level features and markers associated with cervical cancer recurrence and metastasis, offering new potential biomarker targets and a basis for further validation studies.

## CONFLICT OF INTEREST

The authors declare no conflict of interest.

## AUTHOR CONTRIBUTIONS

Conceptualization, SYH, TMK, and YJC; Data curation, JJL, JYH, JEL, JSY, KRK, TMK, and YJC; Formal analysis, JJL and JEL; Funding acquisition, TMK and YJC; Investigation, JJL, JYH, and JEL; Methodology, JJL,DR, and JSY; Project administration, SYH, TMK, and YJC; Resources, JEL, KRK, and SYH; Software, JJL, JSY, and DR; Supervision, SYH, TMK, and YJC; Writing – original draft, JJL; Writing – review & editing, TMKand YJC

## Supporting information

Fig S1

Table S1

Table S2

Table S3

Table S4

Table S5

Table S6

Table S7

Supplementary Material

Supplementary Material

Supplementary Material

Supplementary Material

Supplementary Material

## Data Availability

The data that support the findings of this study are available on request from the corresponding author. The data are not publicly available due to privacy or ethical restrictions.
