# Supplementary figures and images for: Genomic, transcriptomic, and viral integration profiles associated with recurrent/metastatic progression in high‐risk human papillomavirus cervical carcinomas
Source: Cancer Med. 2020 Oct 5;9(21):8243–57. doi: 10.1002/cam4.3426 (PMC7643681; doi:10.1002/cam4.3426)

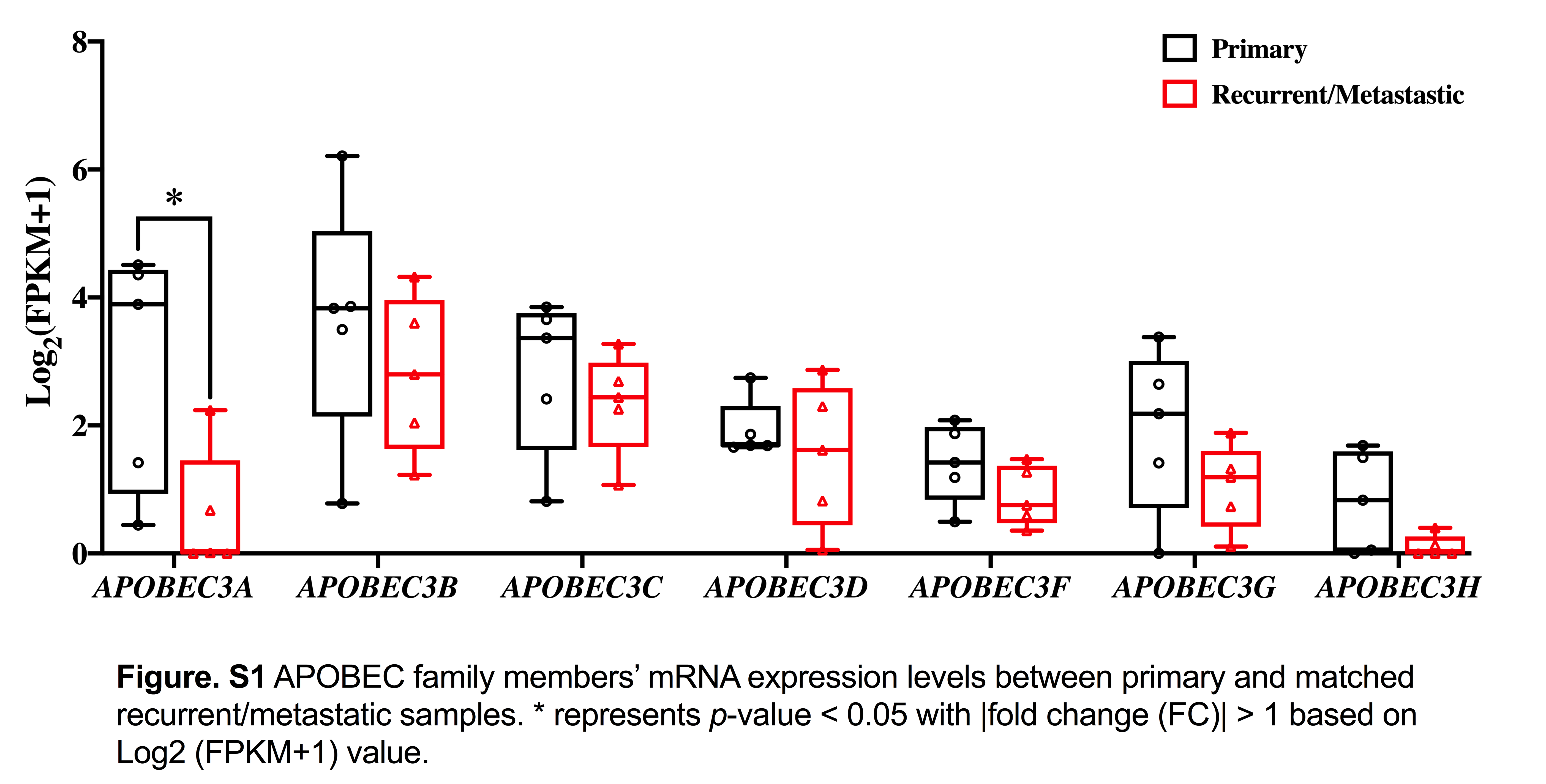

Supplement: Supplementary file 1 — Fig S1 [file CAM4-9-8243-s001.tiff]
