# Supplementary material for: Genomic, transcriptomic, and viral integration profiles associated with recurrent/metastatic progression in high‐risk human papillomavirus cervical carcinomas
Source: Cancer Med. 2020 Oct 5;9(21):8243–57. doi: 10.1002/cam4.3426 (PMC7643681; doi:10.1002/cam4.3426)
Supplement: Supplementary file 9 — Supplementary Material [file CAM4-9-8243-s009.pdf]

MultiQC

(http://multiqc.info)

A modular tool to aggregate results from bioinformatics analyses across many samples into a single report.

Report generated on 2020-06-15, 07:45 based on data in: `/Volumes/sequencing data/Test/DNA RAW`

General Statistics

Copy table | Configure Columns | Plot | Showing 20/20 rows and 3/5 columns.

Sample Name	% Dups	% GC	M Seqs
CES1-P_FFPE_DNA_1	38.2%	50%	62.9
CES1-P_FFPE_DNA_2	29.4%	50%	62.9
CES1-R:M_FFPE_DNA_1	47.9%	51%	60.9
CES1-R:M_FFPE_DNA_2	44.9%	50%	60.9
CES2-P_FFPE_DNA_1	35.3%	52%	63.3
CES2-P_FFPE_DNA_2	33.7%	52%	63.3
CES2-R:M_FFPE_DNA_1	32.3%	52%	68.0
CES2-R:M_FFPE_DNA_2	29.6%	52%	68.0
CES3-P_FFPE_DNA_1	24.9%	53%	59.7
CES3-P_FFPE_DNA_2	18.6%	53%	59.7
CES3-R:M_FFPE_DNA_1	30.9%	52%	62.7
CES3-R:M_FFPE_DNA_2	28.8%	52%	62.7
CES4-P_FFPE_DNA_1	31.5%	55%	84.7
CES4-P_FFPE_DNA_2	28.5%	54%	84.7
CES4-R:M_FFPE_DNA_1	45.3%	48%	86.7
CES4-R:M_FFPE_DNA_2	40.9%	48%	86.7
CES5-P_FFPE_DNA_1	30.0%	54%	83.7
CES5-P_FFPE_DNA_2	26.8%	54%	83.7
CES5-R:M_FFPE_DNA_1	24.2%	53%	60.8
CES5-R:M_FFPE_DNA_2	22.7%	53%	60.8

FastQC

FastQC (http://www.bioinformatics.babraham.ac.uk/projects/fastqc/) is a quality control tool for high throughput sequence data, written by Simon Andrews at the Babraham Institute in Cambridge.

Sequence Counts

Help

Sequence counts for each sample. Duplicate read counts are an estimate only.

Number of reads | Percentages

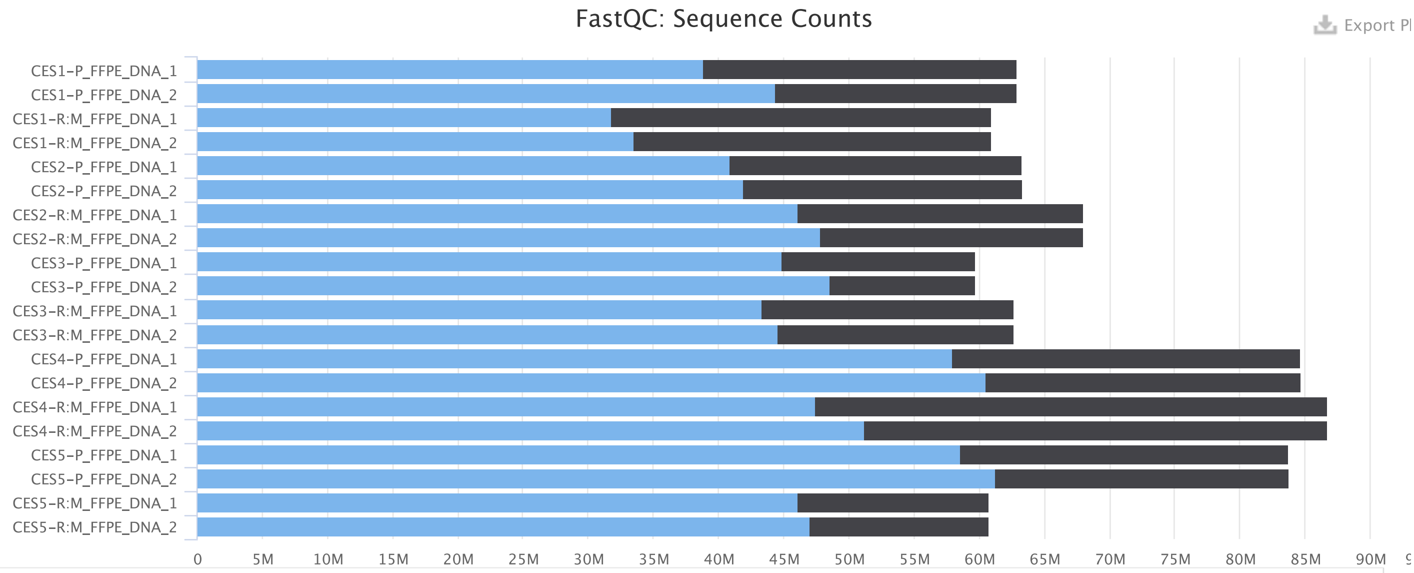

Sequence Quality Histograms 20

Help

The mean quality value across each base position in the read.

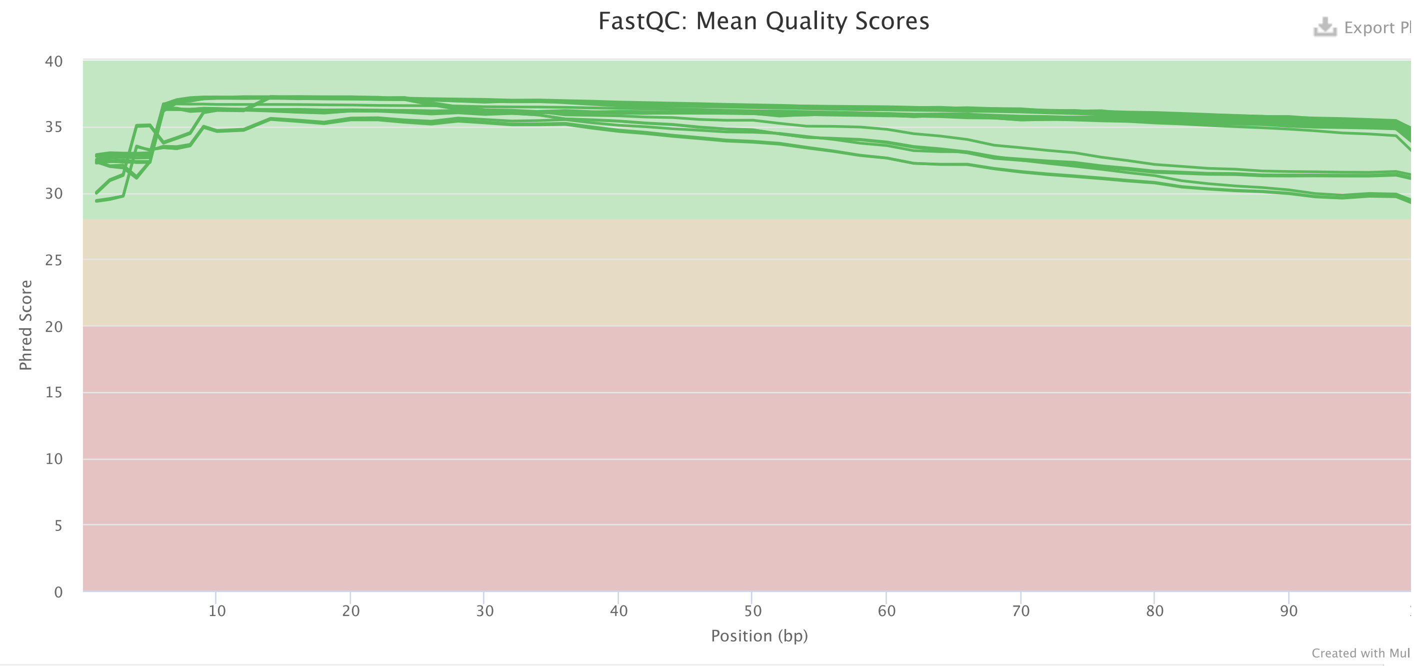

Per Sequence Quality Scores 20

Help

The number of reads with average quality scores. Shows if a subset of reads has poor quality.

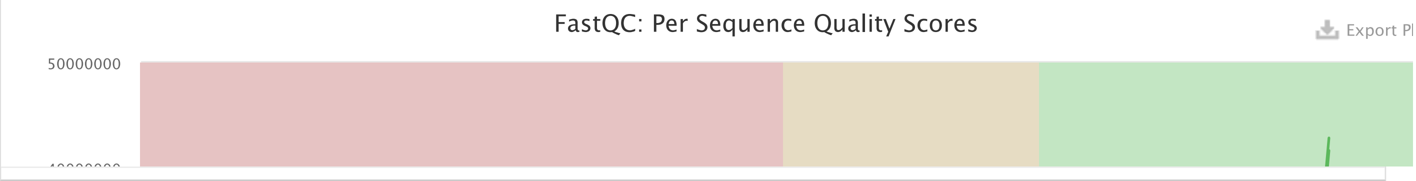

Per Base Sequence Content 20

Help

The proportion of each base position for which each of the four normal DNA bases has been called.

Click a sample row to see a line plot for that dataset.

Rollover for sample name

Position: - %T: - %C: - %A: - %G: -

Export Plot

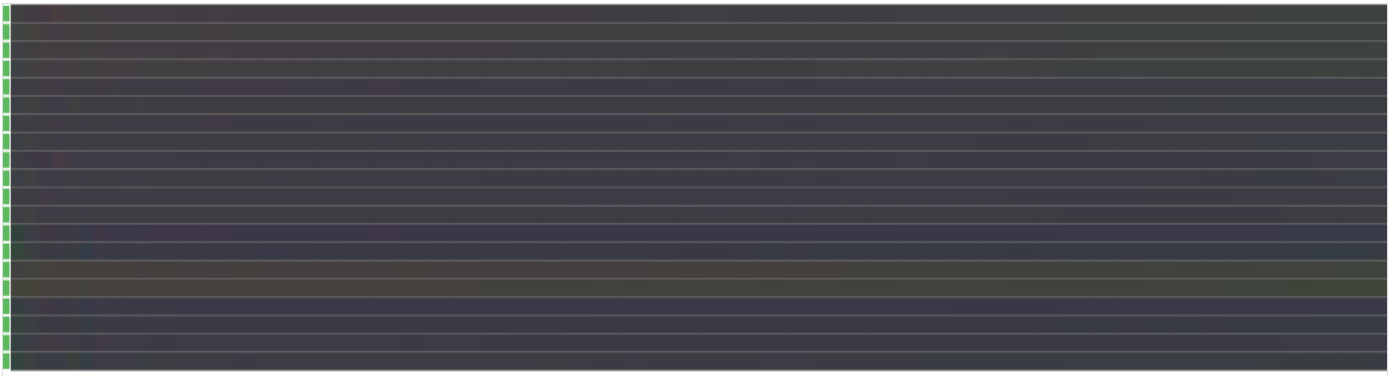

Per Sequence GC Content 0 20

Help

The average GC content of reads. Normal random library typically have a roughly normal distribution of GC content.

Percentages | Counts

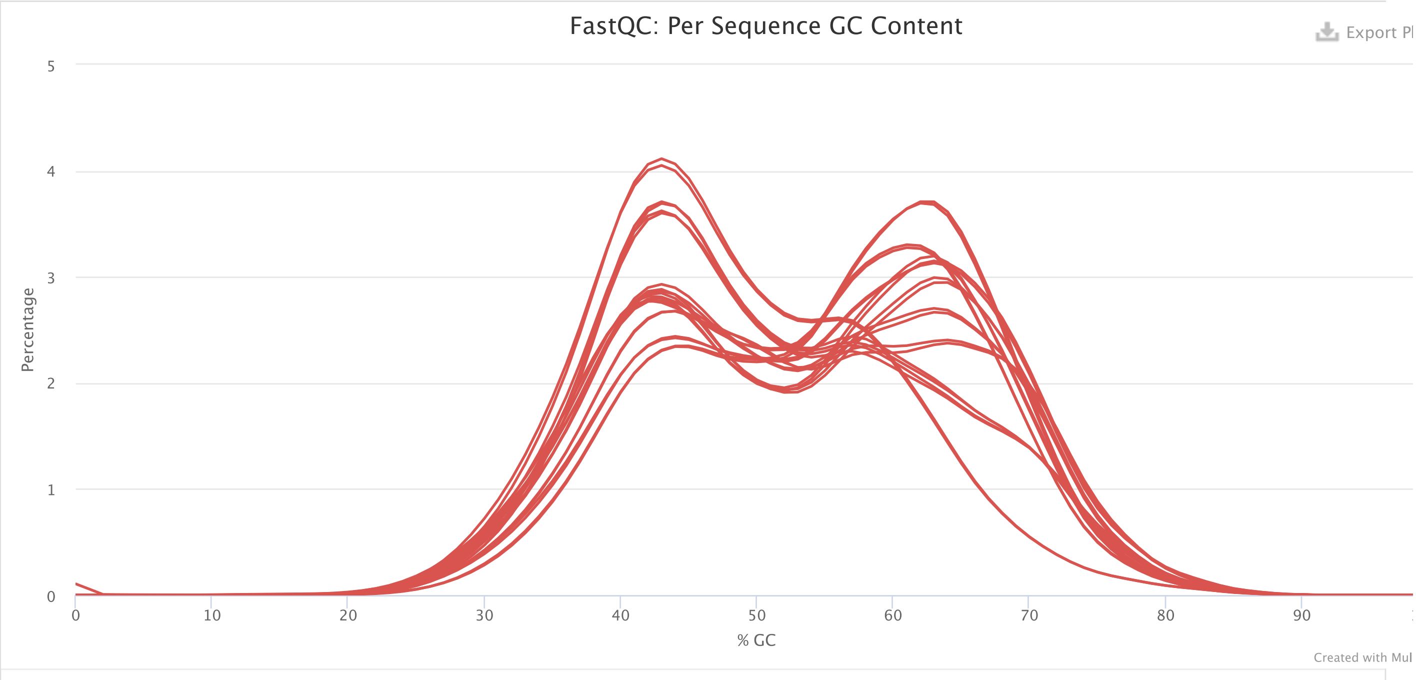

Per Base N Content 20

Help

The percentage of base calls at each position for which an N was called.

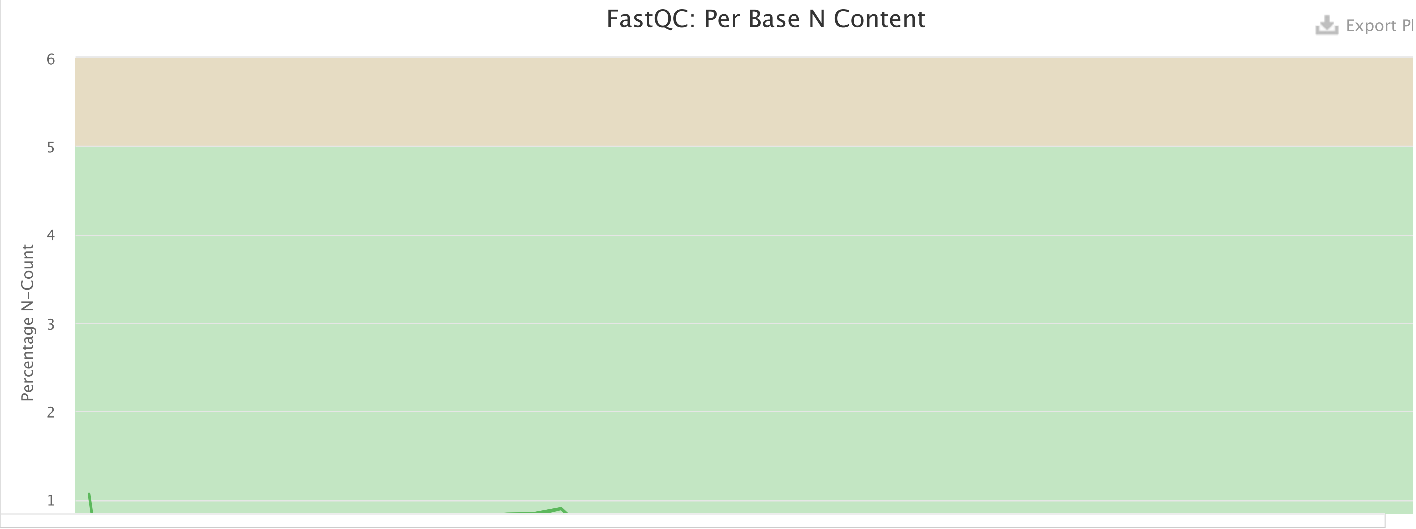

Sequence Length Distribution 20

All samples have sequences of a single length (101bp).

Sequence Duplication Levels 9 11

Help

The relative level of duplication found for every sequence.

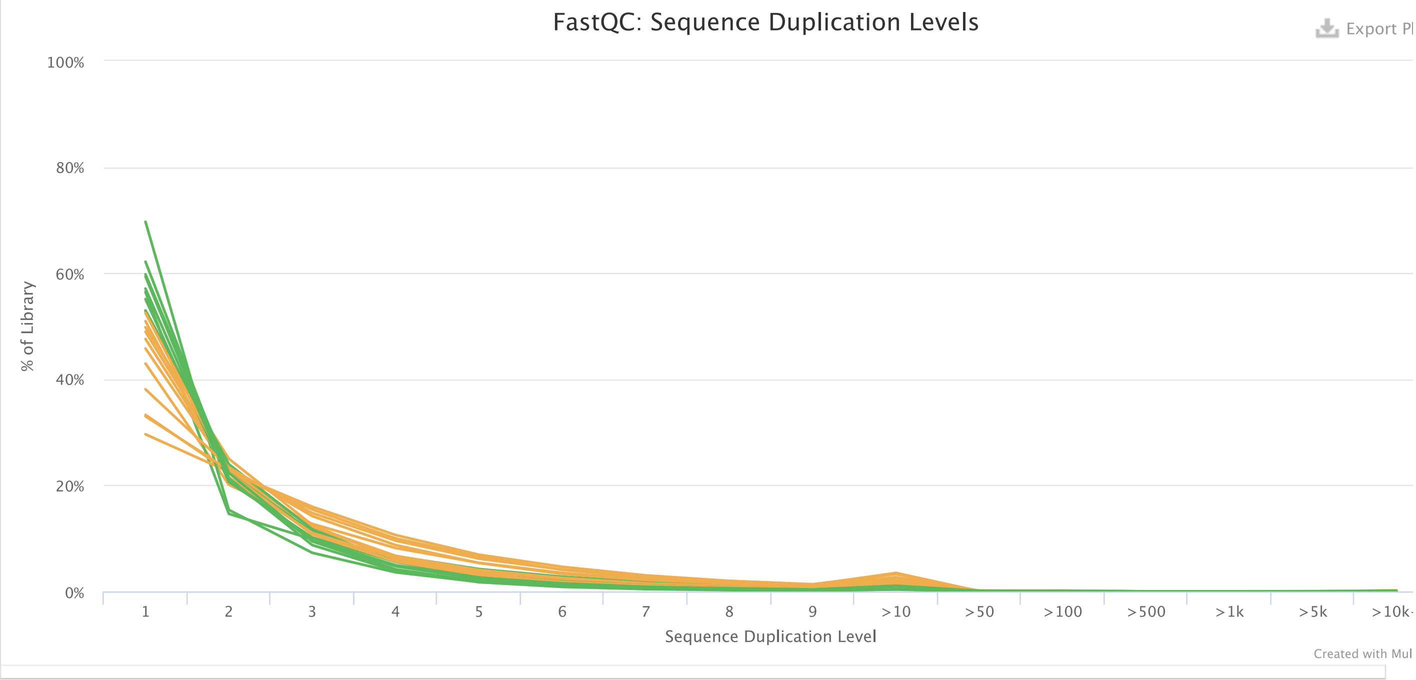

Overrepresented sequences 17 3

Help

The total amount of overrepresented sequences found in each library.

20 samples had less than 1% of reads made up of overrepresented sequences

Adapter Content 18 2

Help

The cumulative percentage count of the proportion of your library which has seen each of the adapter sequences at each position.

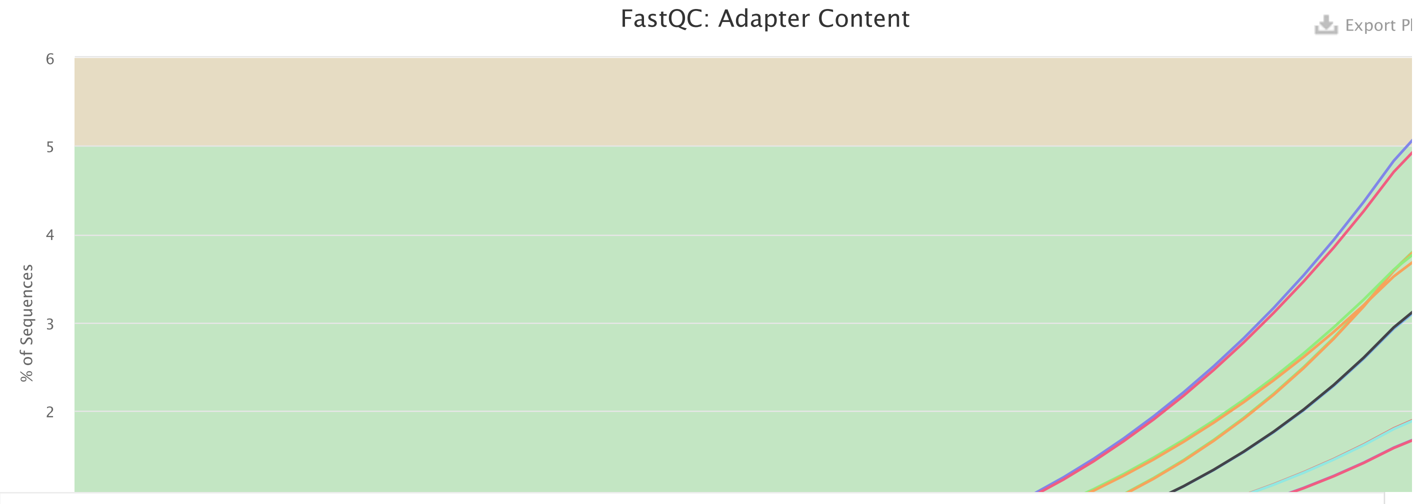

Status Checks

Help

Status for each FastQC section showing whether results seem entirely normal (green), slightly abnormal (orange) or very unusual (red).

Sort by highlight

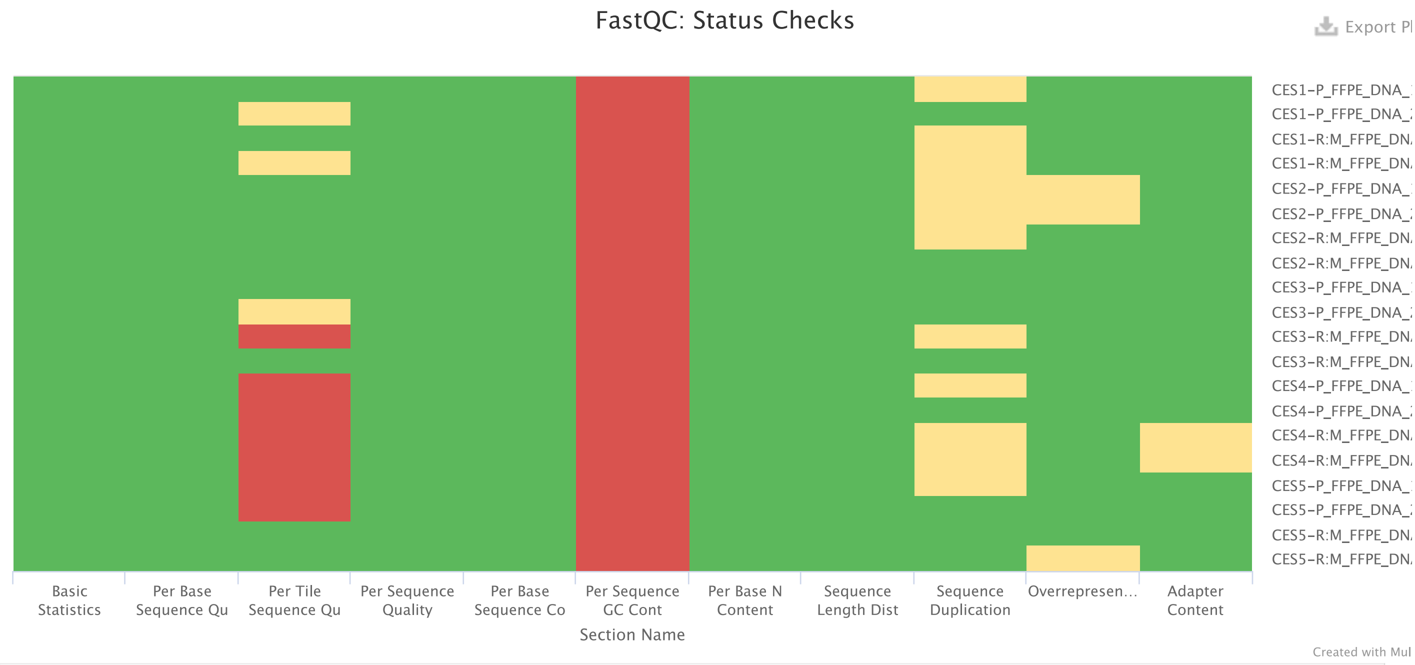

MultiQC v1.9 (http://multiqc.info) - Written by Phil Ewels (http://phil.ewels.co.uk), available on GitHub (https://github.com/ewels/MultiQC).
This report uses HighCharts (http://www.highcharts.com/), jQuery (https://jquery.com/), jQuery UI (https://jqueryui.com/), Bootstrap (http://getbootstrap.com/), FileSaver.js (https://github.com/eligrey/FileSaver.js) and clipboard.js (https://clipboardjs.com/).

SciLifeLab (http://www.scilifelab.se/)
