# Supplementary material for: Genomic, transcriptomic, and viral integration profiles associated with recurrent/metastatic progression in high‐risk human papillomavirus cervical carcinomas
Source: Cancer Med. 2020 Oct 5;9(21):8243–57. doi: 10.1002/cam4.3426 (PMC7643681; doi:10.1002/cam4.3426)
Supplement: Supplementary file 10 — Supplementary Material [file CAM4-9-8243-s010.pdf]

(http://multiqc.info)

A modular tool to aggregate results from bioinformatics analyses across many samples into a single report.

Report generated on 2020-06-15, 07:48 based on data in: `/Volumes/sequencing data/Test/DNA Bam`

General Statistics

Copy table | Configure Columns | Plot | Showing 10/10 rows and 3/5 columns.

Sample Name	% Dups	% GC	M Seqs
CES1-P_FFPE_DNA.dupRemove.realign.fix.recal	14.9%	50%	75.5
CES1-R:M_FFPE_DNA.dupRemove.realign.fix.recal	16.0%	50%	73.6
CES2-P_FFPE_DNA.dupRemove.realign.fix.recal	21.8%	52%	97.7
CES2-R:M_FFPE_DNA.dupRemove.realign.fix.recal	24.2%	52%	117.3
CES3-P_FFPE_DNA.dupRemove.realign.fix.recal	22.2%	53%	101.9
CES3-R:M_FFPE_DNA.dupRemove.realign.fix.recal	27.2%	52%	114.5
CES4-P_FFPE_DNA.dupRemove.realign.fix.recal	17.3%	54%	152.0
CES4-R:M_FFPE_DNA.dupRemove.realign.fix.recal	12.8%	48%	108.9
CES5-P_FFPE_DNA.dupRemove.realign.fix.recal	18.9%	54%	154.1
CES5-R:M_FFPE_DNA.dupRemove.realign.fix.recal	18.8%	53%	113.3

FastQC

FastQC (http://www.bioinformatics.babraham.ac.uk/projects/fastqc/) is a quality control tool for high throughput sequence data, written by Simon Andrews at the Babraham Institute in Cambridge.

Sequence Counts

Help

Sequence counts for each sample. Duplicate read counts are an estimate only.

Number of reads | Percentages

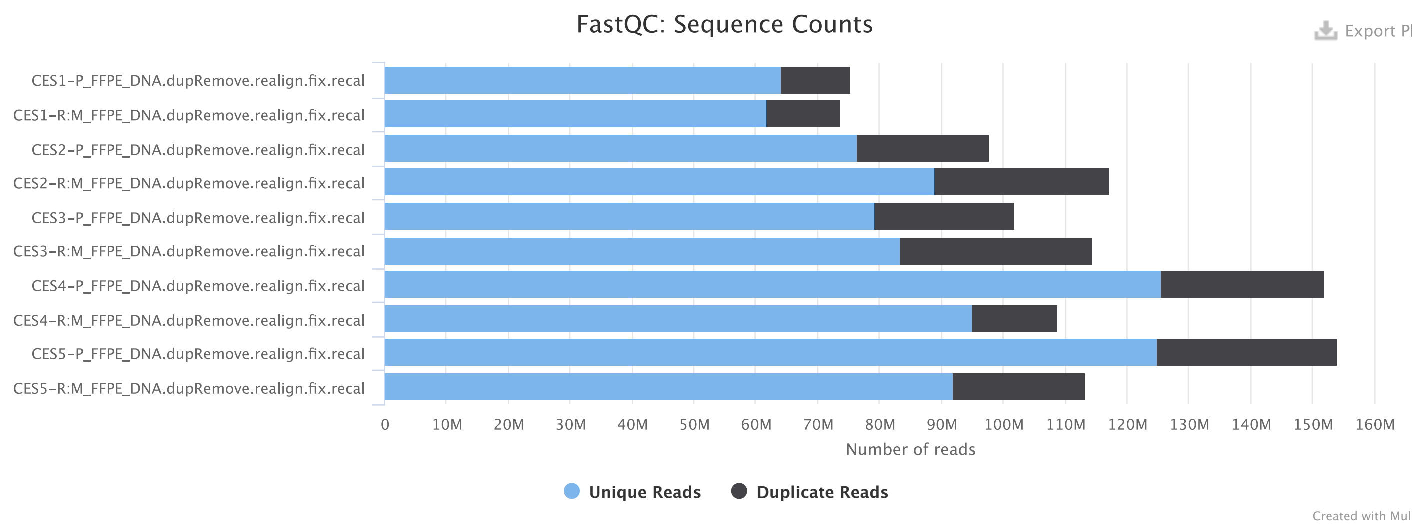

Sequence Quality Histograms 9 1

Help

The mean quality value across each base position in the read.

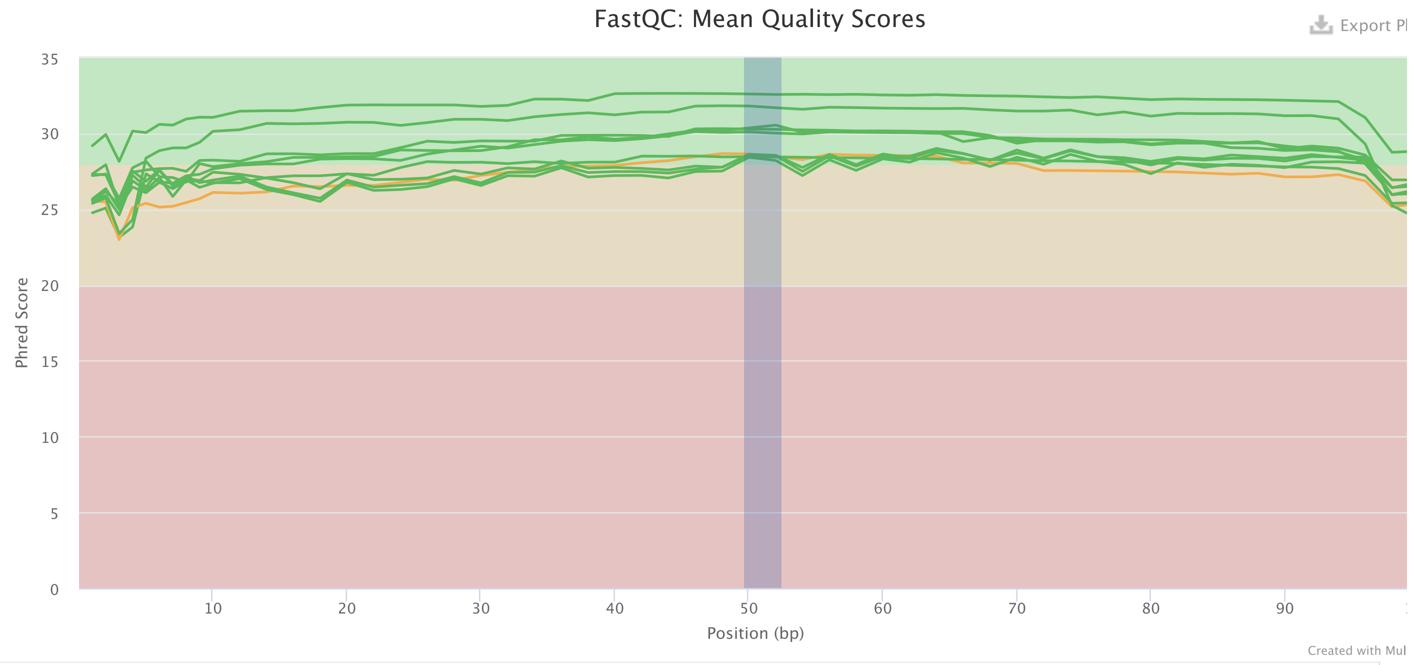

Per Sequence Quality Scores 9 1

Help

The number of reads with average quality scores. Shows if a subset of reads has poor quality.

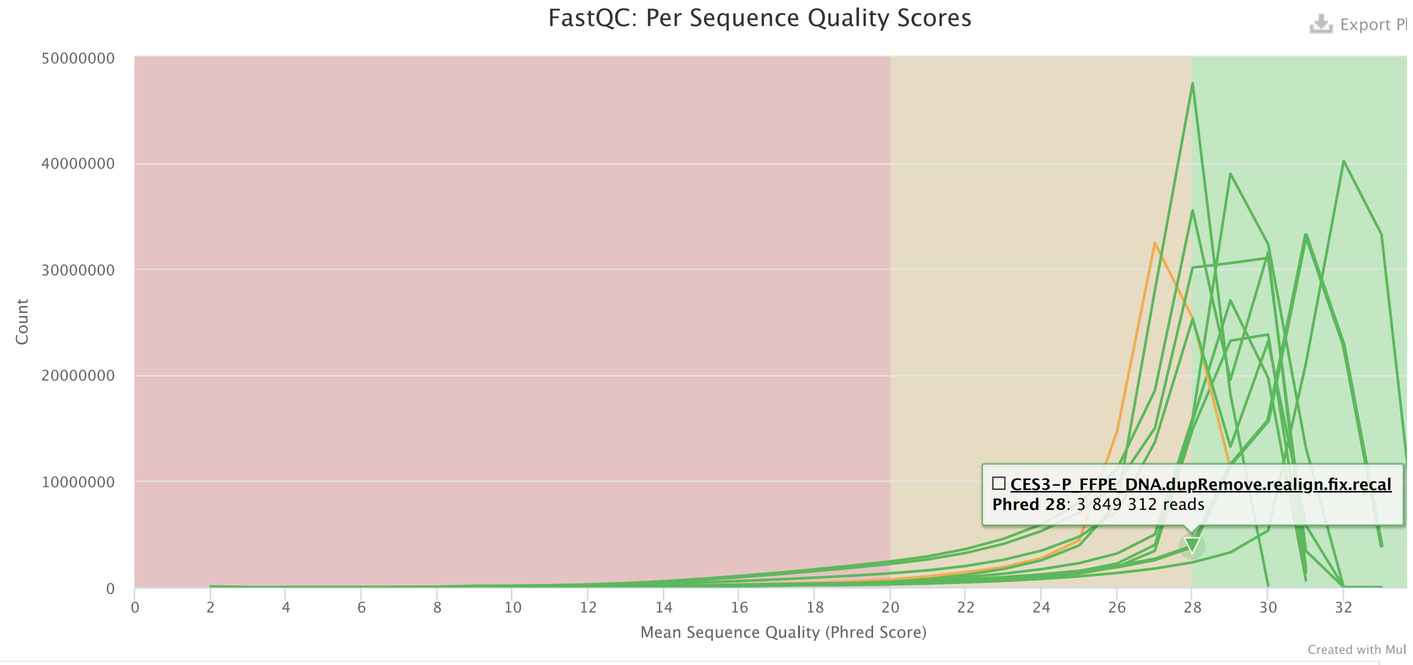

Per Base Sequence Content 10

Help

The proportion of each base position for which each of the four normal DNA bases has been called.

Click a sample row to see a line plot for that dataset.

Rollover for sample name

Position: - %T: - %C: - %A: - %G: -

Export Plot

[redacted]

The average GC content of reads. Normal random library typically have a roughly normal distribution of GC content.

Percentages | Counts

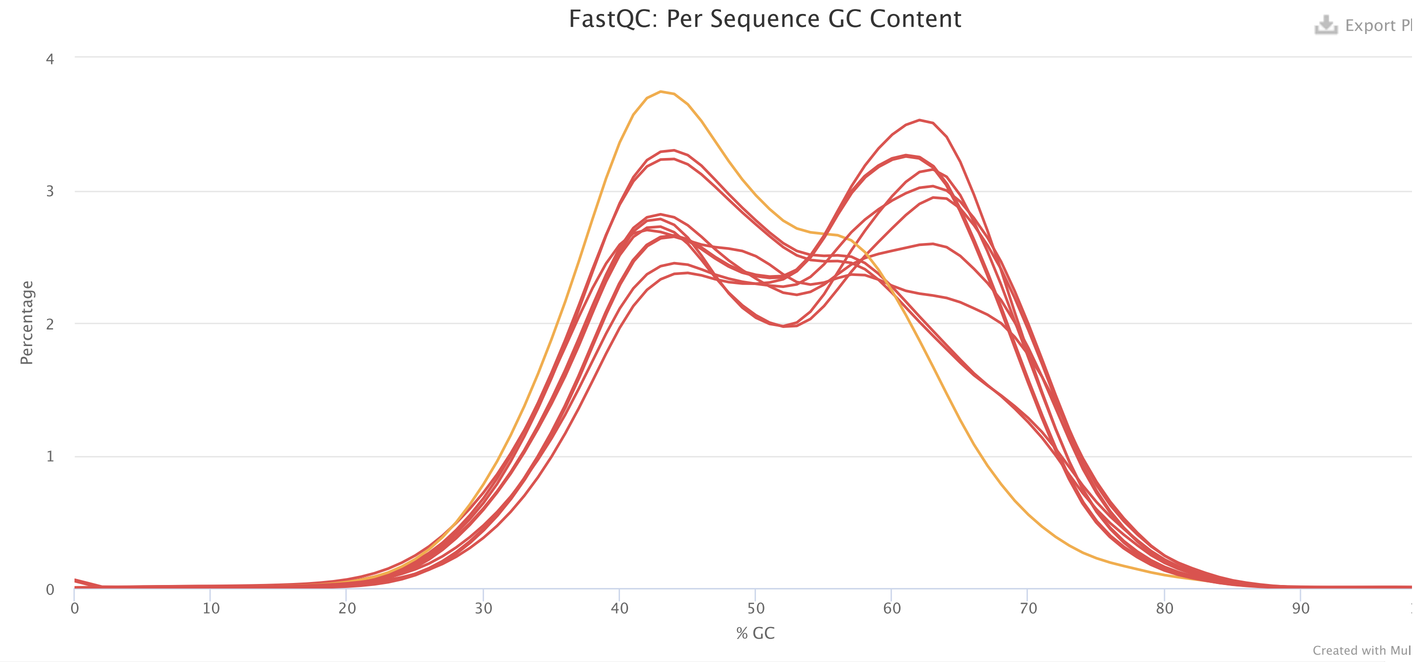

Per Base N Content 10

Help

The percentage of base calls at each position for which an `N` was called.

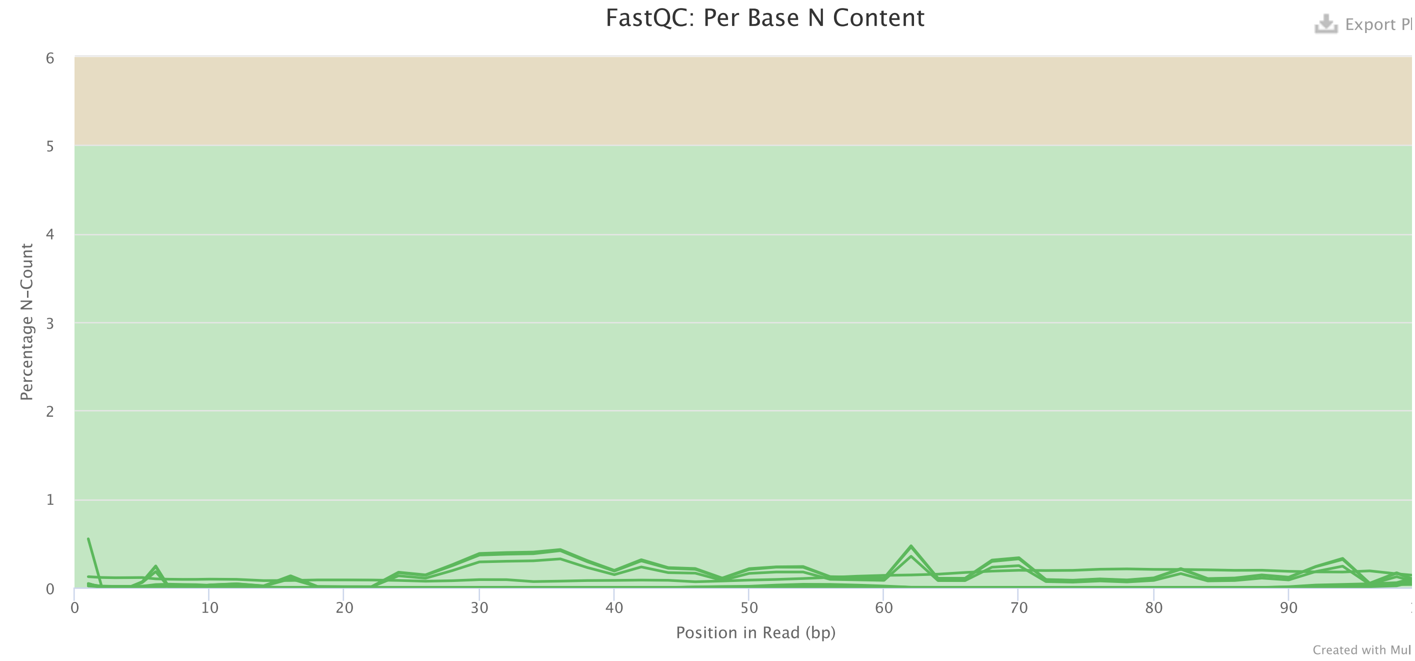

Sequence Length Distribution 0 10

The distribution of fragment sizes (read lengths) found. See the FastQC help (http://www.bioinformatics.babraham.ac.uk/projects/fastqc/Help/3%20Analysis%20Modules/7%20Sequence%20Length%20Distribution.html)

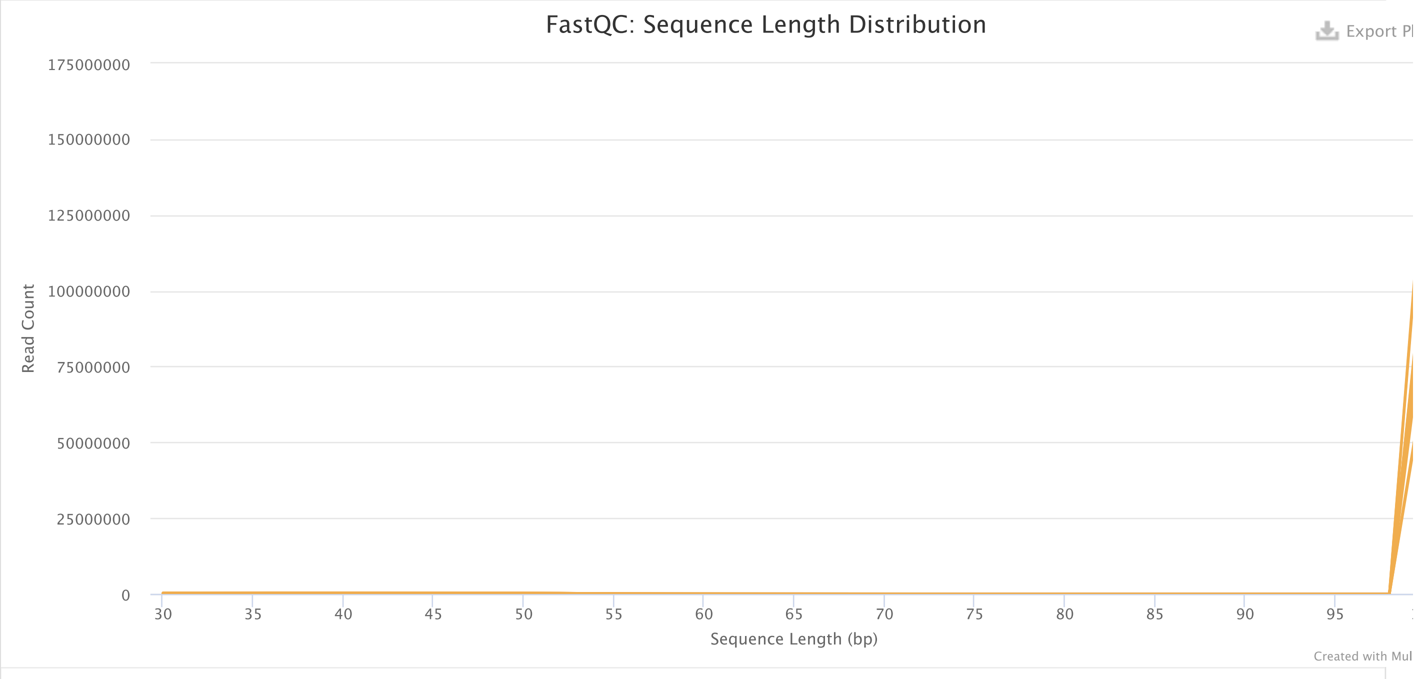

Sequence Duplication Levels 10

Help

The relative level of duplication found for every sequence.

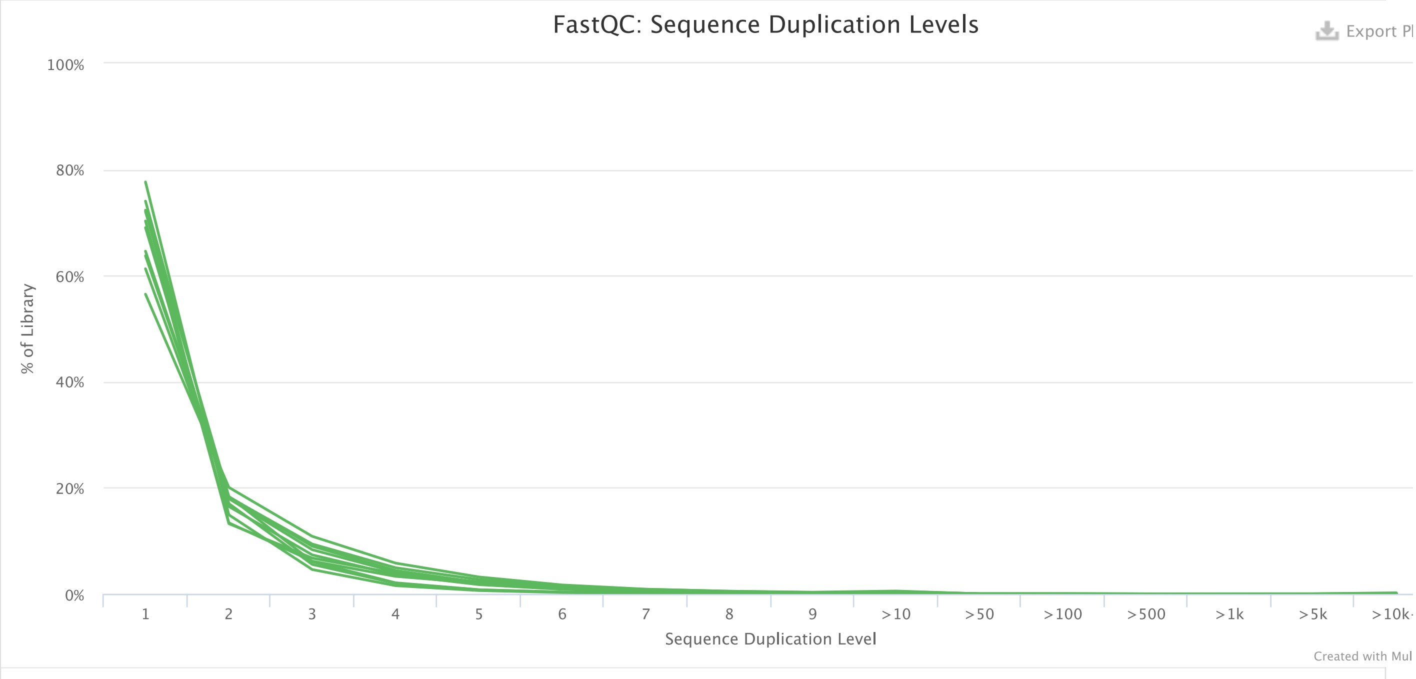

Overrepresented sequences 9 1

Help

The total amount of overrepresented sequences found in each library.

10 samples had less than 1% of reads made up of overrepresented sequences

Adapter Content 9 1

Help

The cumulative percentage count of the proportion of your library which has seen each of the adapter sequences at each position.

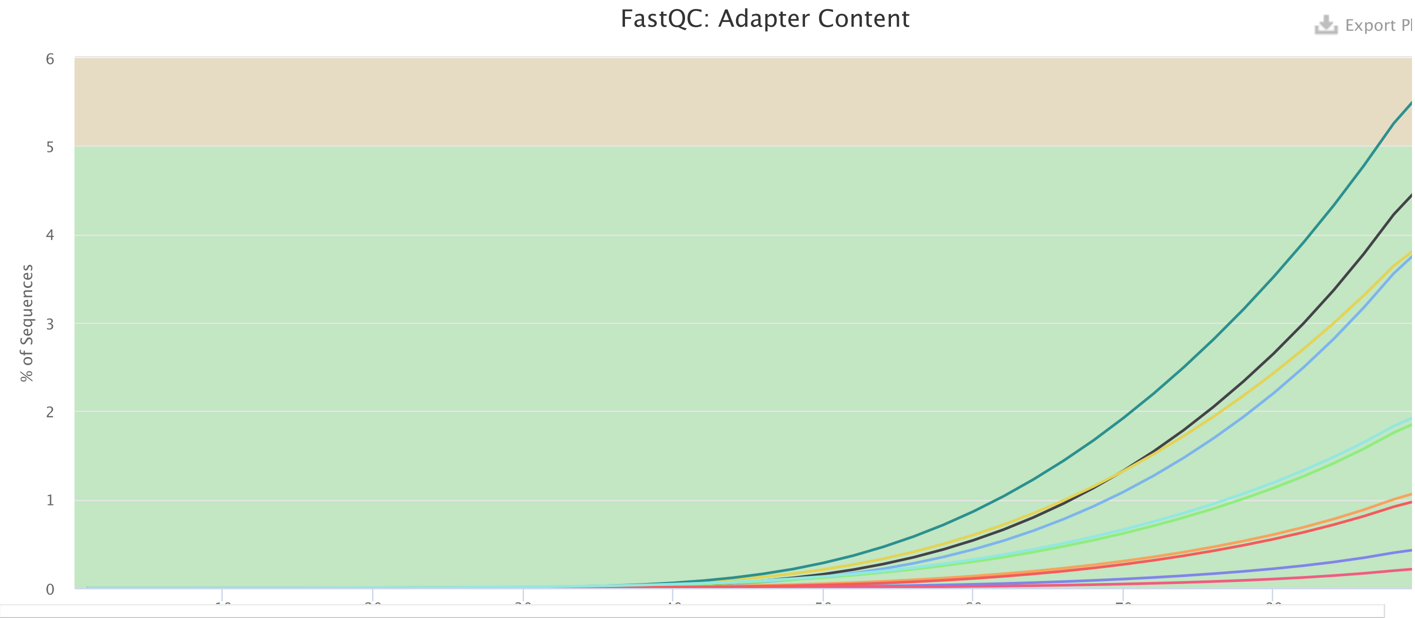

Status Checks

Help

Status for each FastQC section showing whether results seem entirely normal (green), slightly abnormal (orange) or very unusual (red).

Sort by highlight

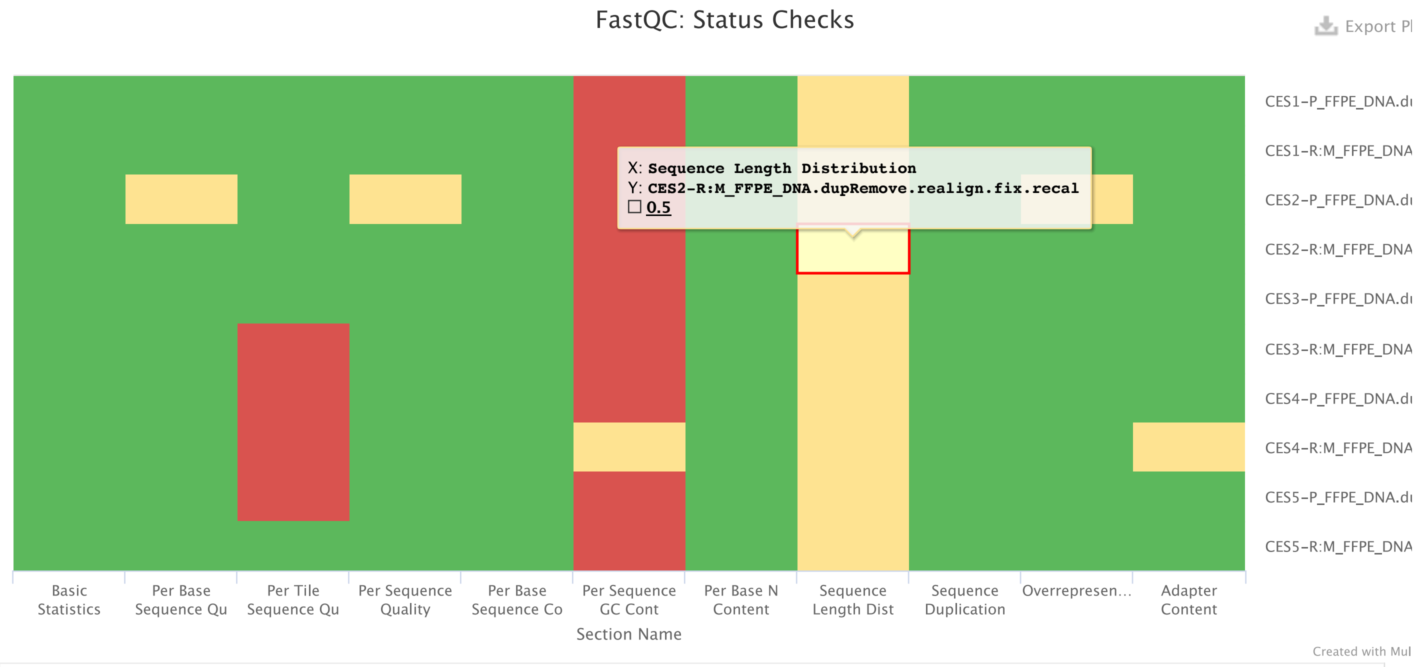

MultiQC v1.9 (http://multiqc.info) - Written by Phil Ewels (http://phil.ewels.co.uk), available on GitHub (https://github.com/ewels/MultiQC).
This report uses HighCharts (http://www.highcharts.com/), jQuery (https://jquery.com/), jQuery UI (https://jqueryui.com/), Bootstrap (http://getbootstrap.com/), FileSaver.js (https://github.com/eligrey/FileSaver.js) and clipboard.js (https://clipboardjs.com/).

SciLifeLab (http://www.scilifelab.se/)
