# Supplementary material for: Genomic, transcriptomic, and viral integration profiles associated with recurrent/metastatic progression in high‐risk human papillomavirus cervical carcinomas
Source: Cancer Med. 2020 Oct 5;9(21):8243–57. doi: 10.1002/cam4.3426 (PMC7643681; doi:10.1002/cam4.3426)
Supplement: Supplementary file 11 — Supplementary Material [file CAM4-9-8243-s011.pdf]

(http://multiqc.info)

A modular tool to aggregate results from bioinformatics analyses across many samples into a single report.

Report generated on 2020-06-15, 03:18 based on data in: `/Users/ashley/Desktop/text/RNA/Before`

General Statistics

Copy table | Configure Columns | Plot | Showing 20/20 rows and 3/5 columns.

Sample Name	% Dups	% GC	M Seqs
CES1_P_RNA_1_fq	97.1%	52%	37.9
CES1_P_RNA_2_fq	92.3%	52%	37.9
CES1_R_M_RNA_1_fq	77.6%	50%	34.0
CES1_R_M_RNA_2_fq	91.9%	50%	34.0
CES2_P_RNA_1_fq	80.0%	52%	34.9
CES2_P_RNA_2_fq	89.2%	52%	34.9
CES2_R_M_RNA_1_fq	92.8%	52%	50.0
CES2_R_M_RNA_2_fq	89.7%	52%	50.0
CES3_P_RNA_1_fq	92.6%	52%	39.4
CES3_P_RNA_2_fq	90.4%	52%	39.4
CES3_R_M_RNA_1_fq	91.9%	51%	49.8
CES3_R_M_RNA_2_fq	89.5%	51%	49.8
CES4_P_RNA_1_fq	96.8%	49%	35.7
CES4_P_RNA_2_fq	95.9%	48%	35.7
CES4_R_M_RNA_1_fq	96.9%	48%	29.2
CES4_R_M_RNA_2_fq	96.1%	47%	29.2
CES5_P_RNA_1_fq	96.7%	38%	36.3
CES5_P_RNA_2_fq	96.2%	36%	36.3
CES5_R_M_RNA_1_fq	97.1%	48%	35.1
CES5_R_M_RNA_2_fq	95.3%	48%	35.1

FastQC

FastQC (http://www.bioinformatics.babraham.ac.uk/projects/fastqc/) is a quality control tool for high throughput sequence data, written by Simon Andrews at the Babraham Institute in Cambridge.

Sequence Counts

Help

Sequence counts for each sample. Duplicate read counts are an estimate only.

Number of reads | Percentages

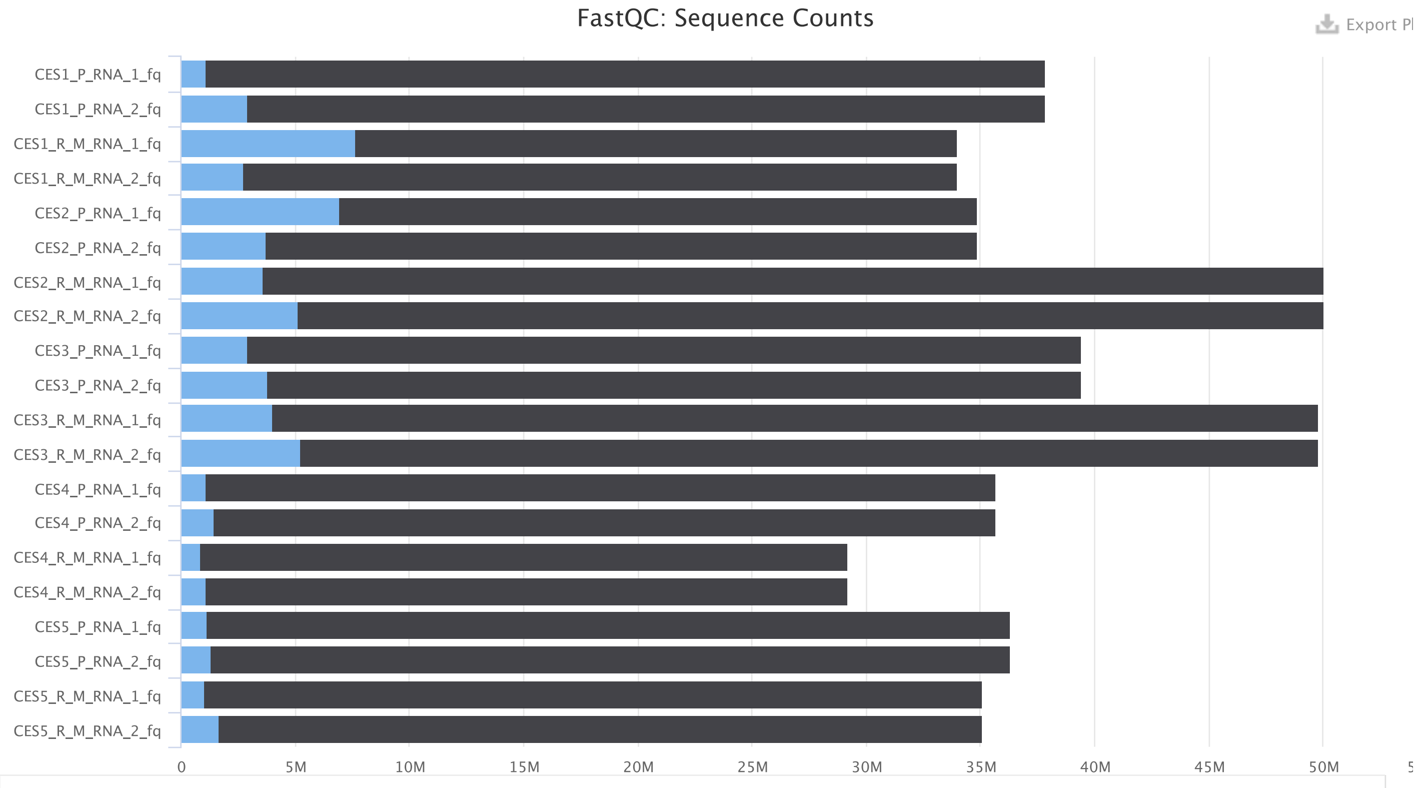

Sequence Quality Histograms 20

Help

The mean quality value across each base position in the read.

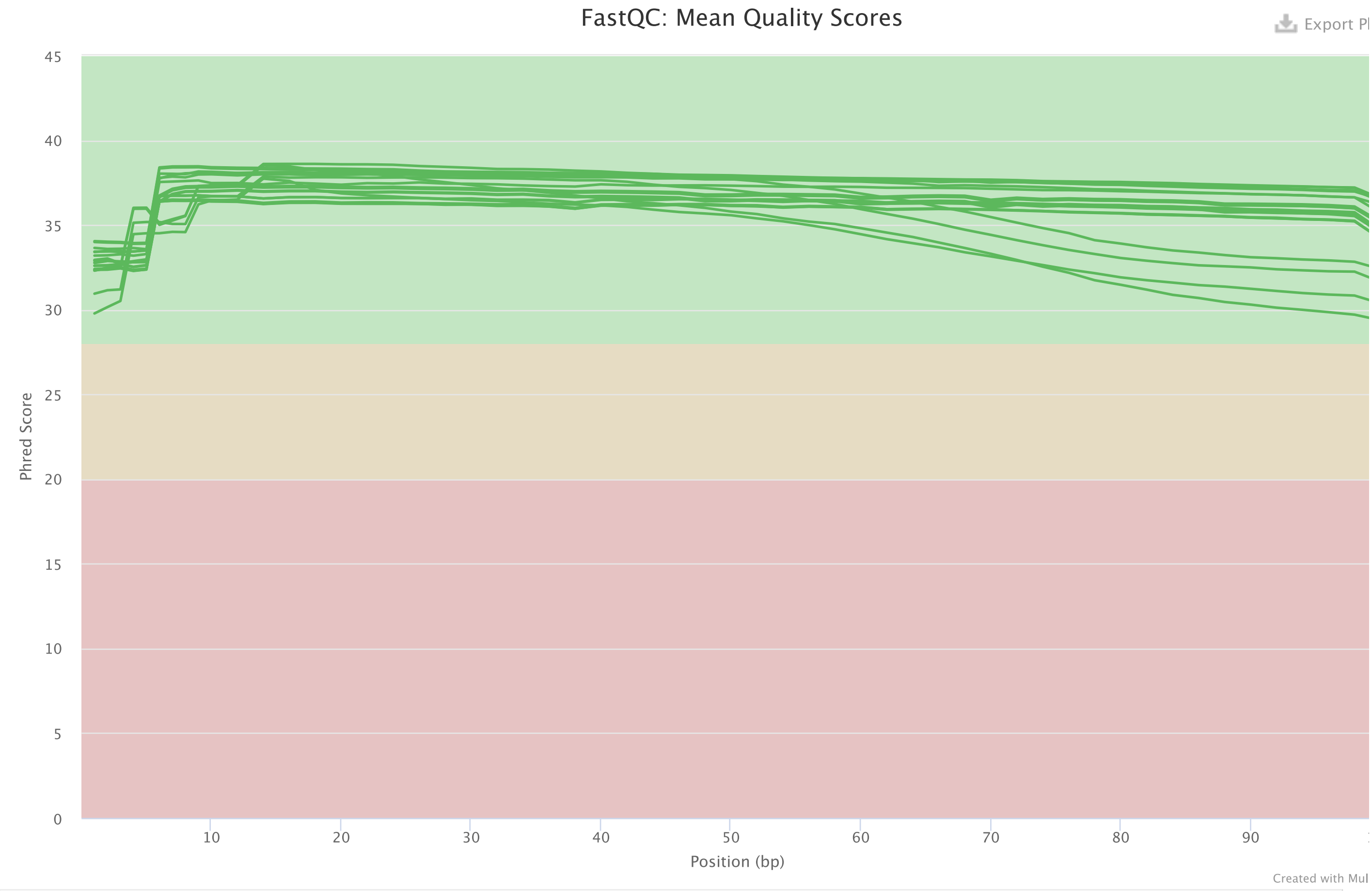

Per Sequence Quality Scores 20

Help

The number of reads with average quality scores. Shows if a subset of reads has poor quality.

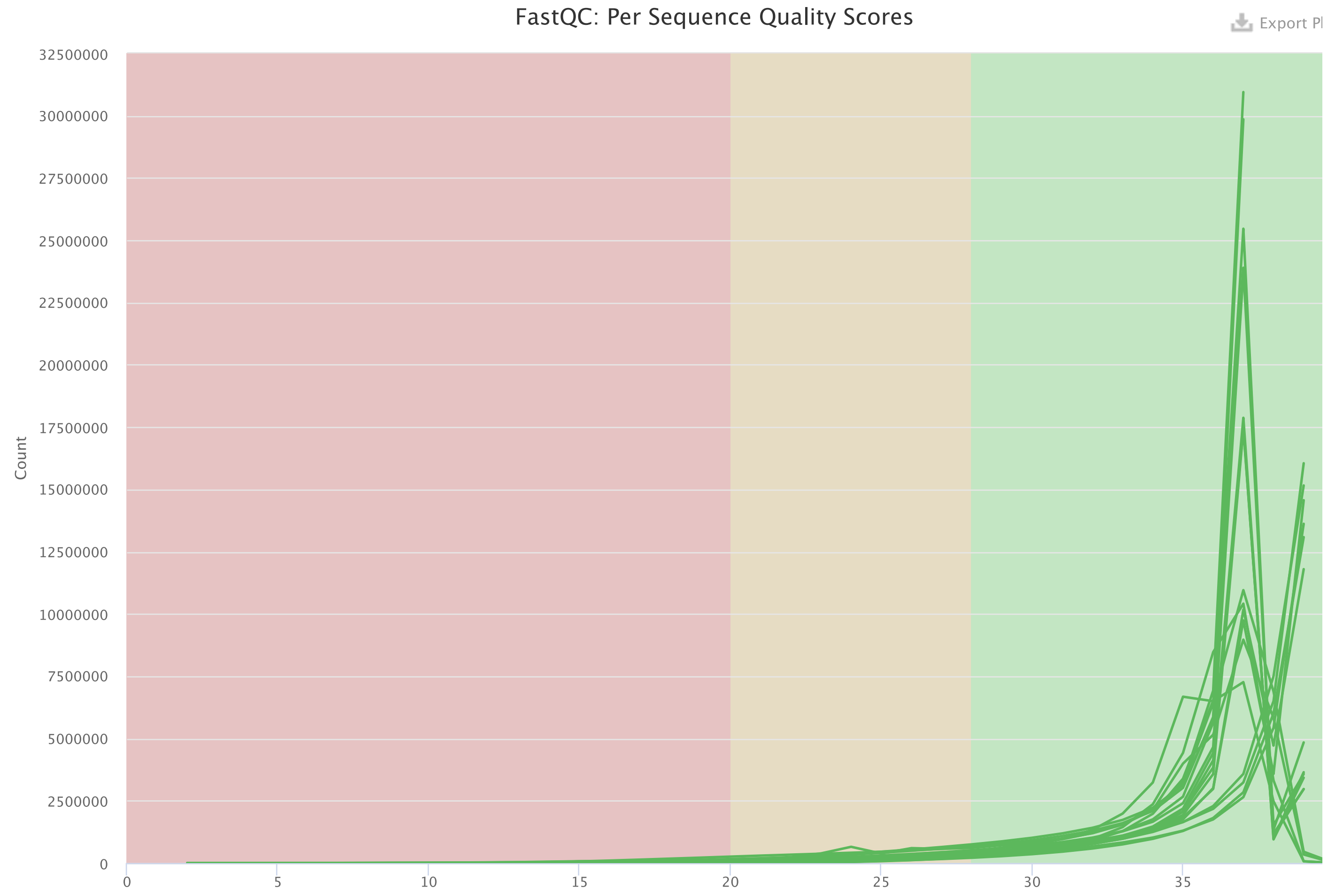

Per Base Sequence Content 0 6 14

Help

The proportion of each base position for which each of the four normal DNA bases has been called.

Click a sample row to see a line plot for that dataset.

Rollover for sample name

Position: - %T: - %C: - %A: - %G: -

Export Plot

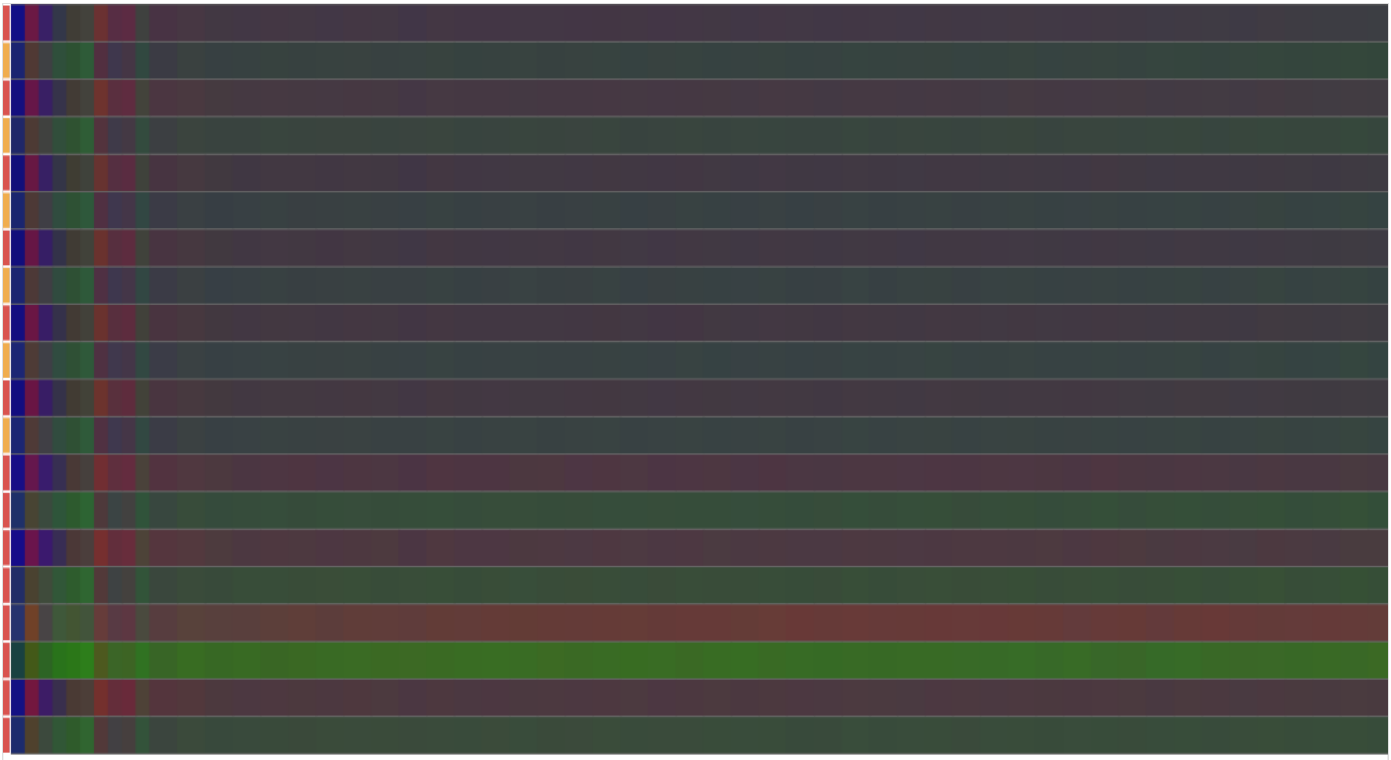

Per Sequence GC Content 4 8 8

Help

The average GC content of reads. Normal random library typically have a roughly normal distribution of GC content.

Percentages Counts

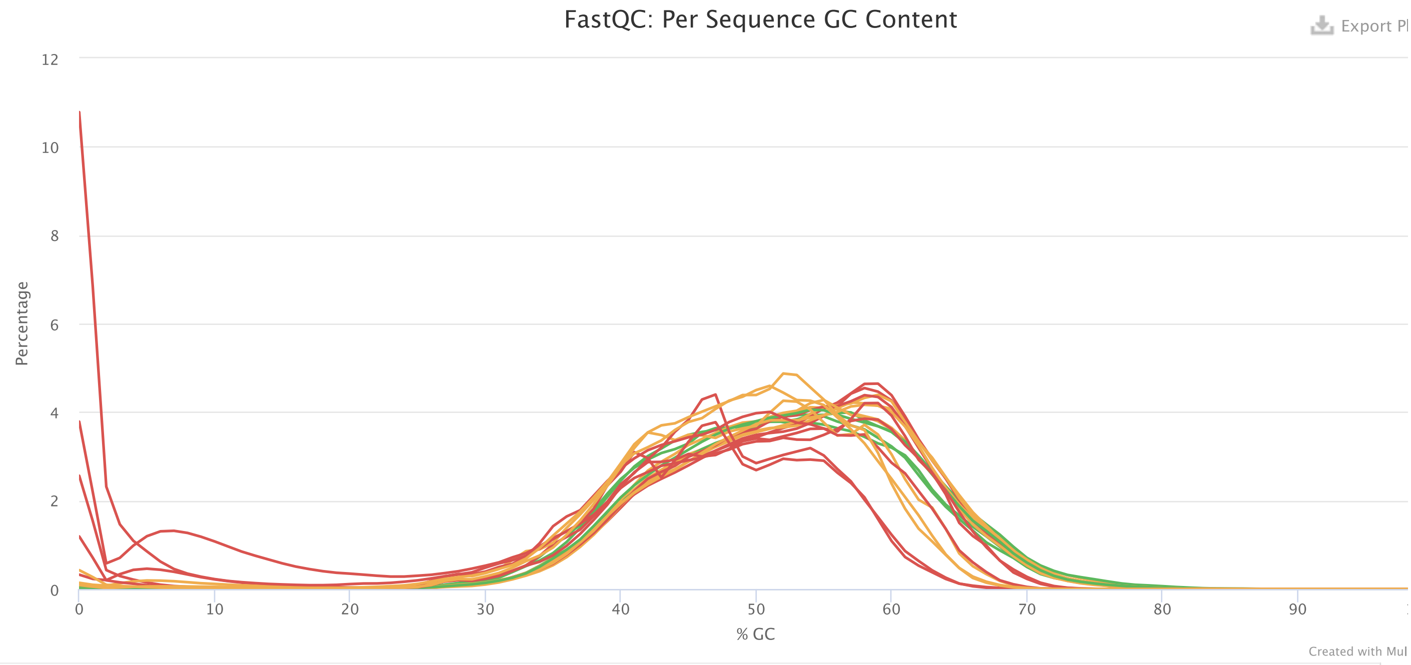

Per Base N Content 20

Help

The percentage of base calls at each position for which an N was called.

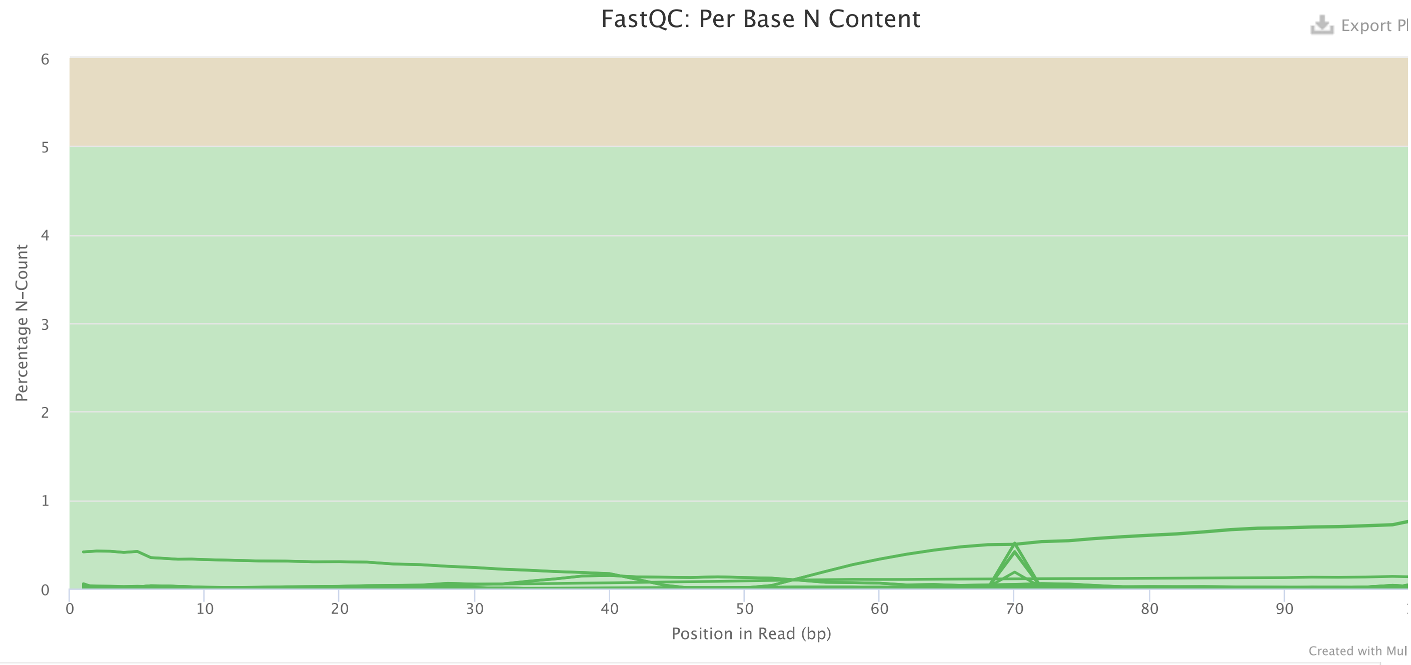

Sequence Length Distribution 20

All samples have sequences of a single length (101bp).

Sequence Duplication Levels 0 20

Help

The relative level of duplication found for every sequence.

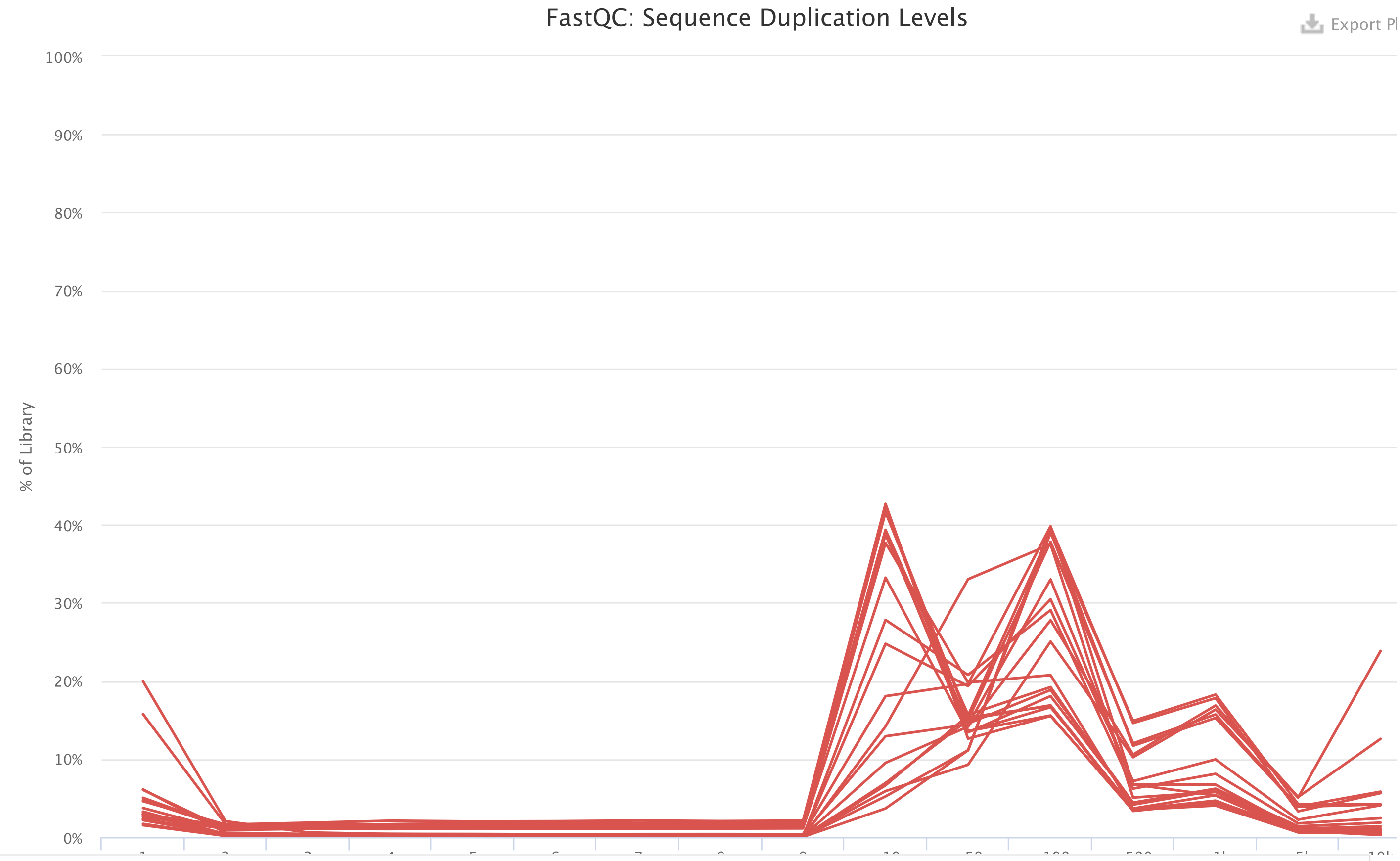

Overrepresented sequences

5 11 4

Help

The total amount of overrepresented sequences found in each library.

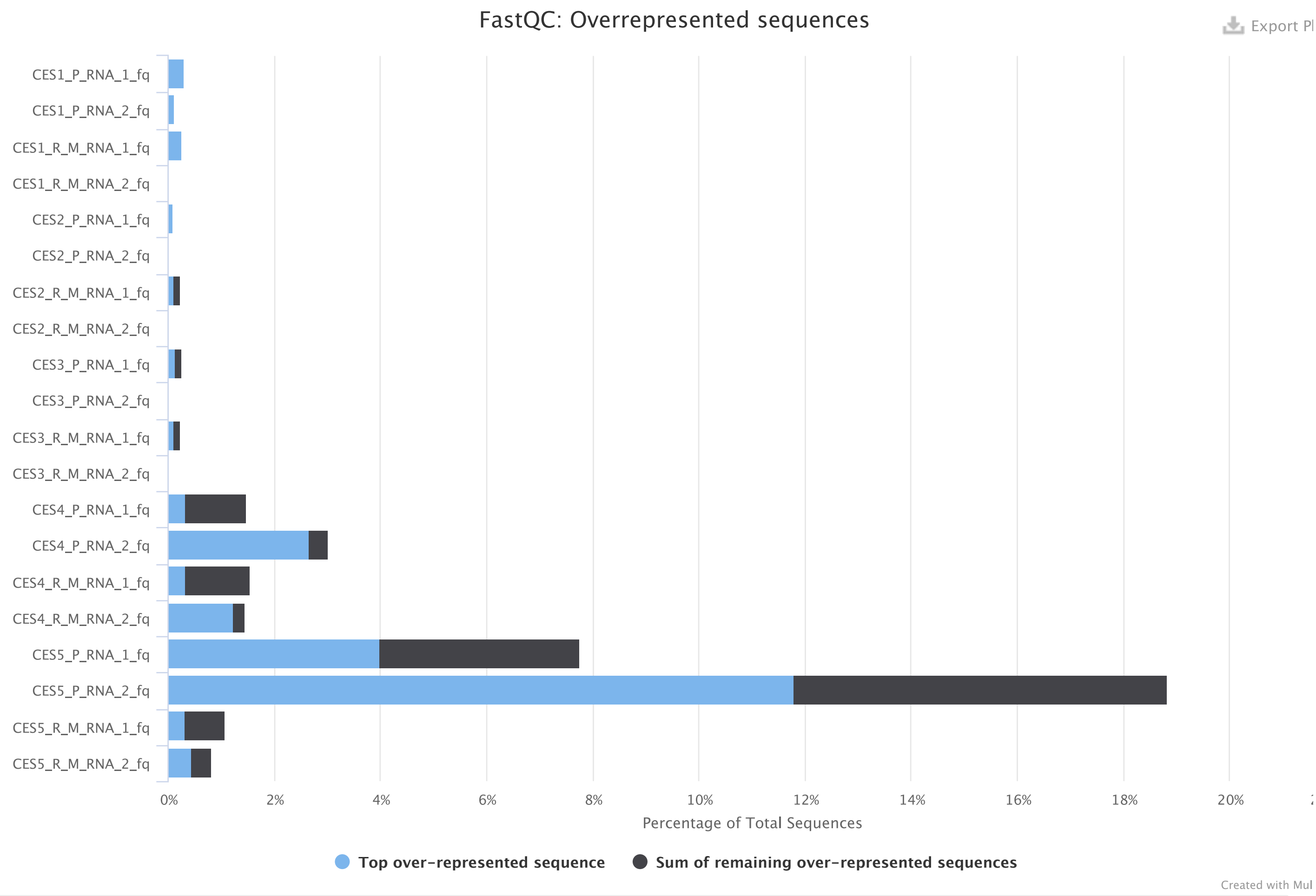

Adapter Content

18 2

Help

The cumulative percentage count of the proportion of your library which has seen each of the adapter sequences at each position.

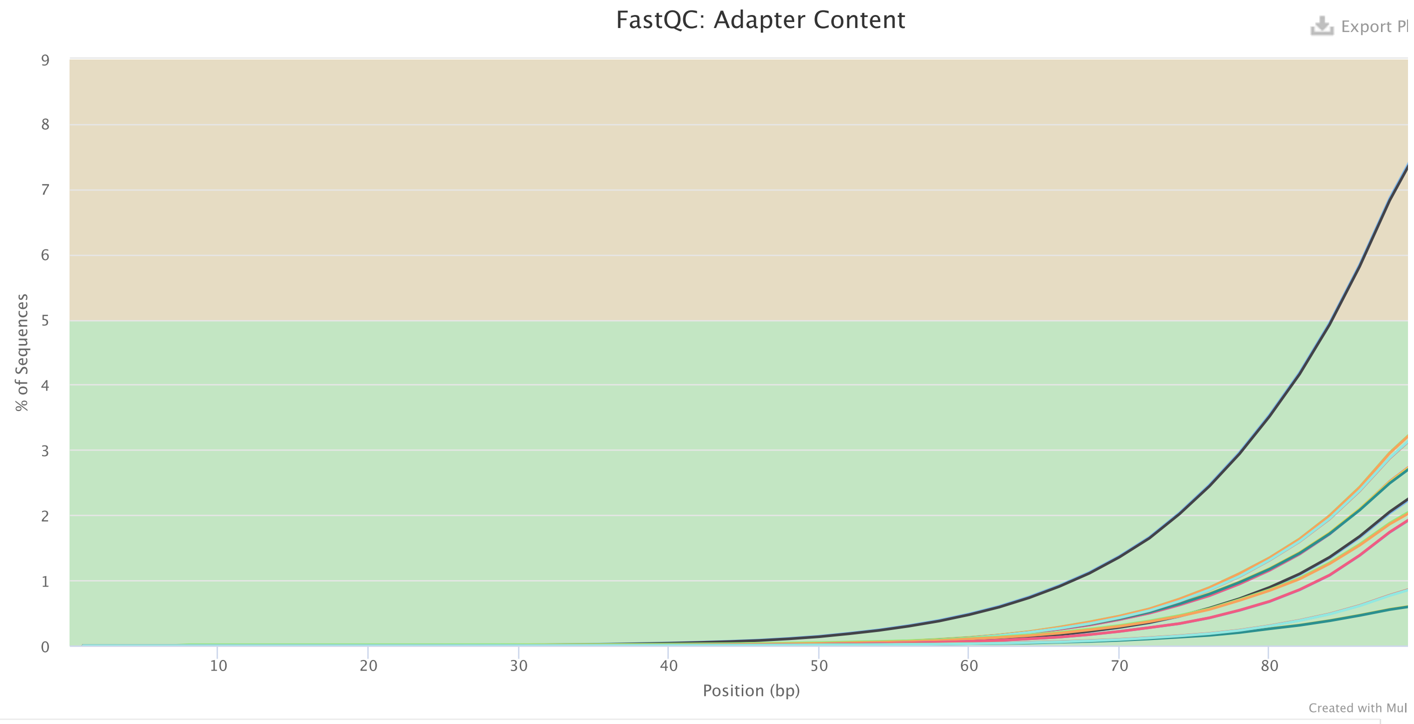

Status Checks

Help

Status for each FastQC section showing whether results seem entirely normal (green), slightly abnormal (orange) or very unusual (red).

Sort by highlight

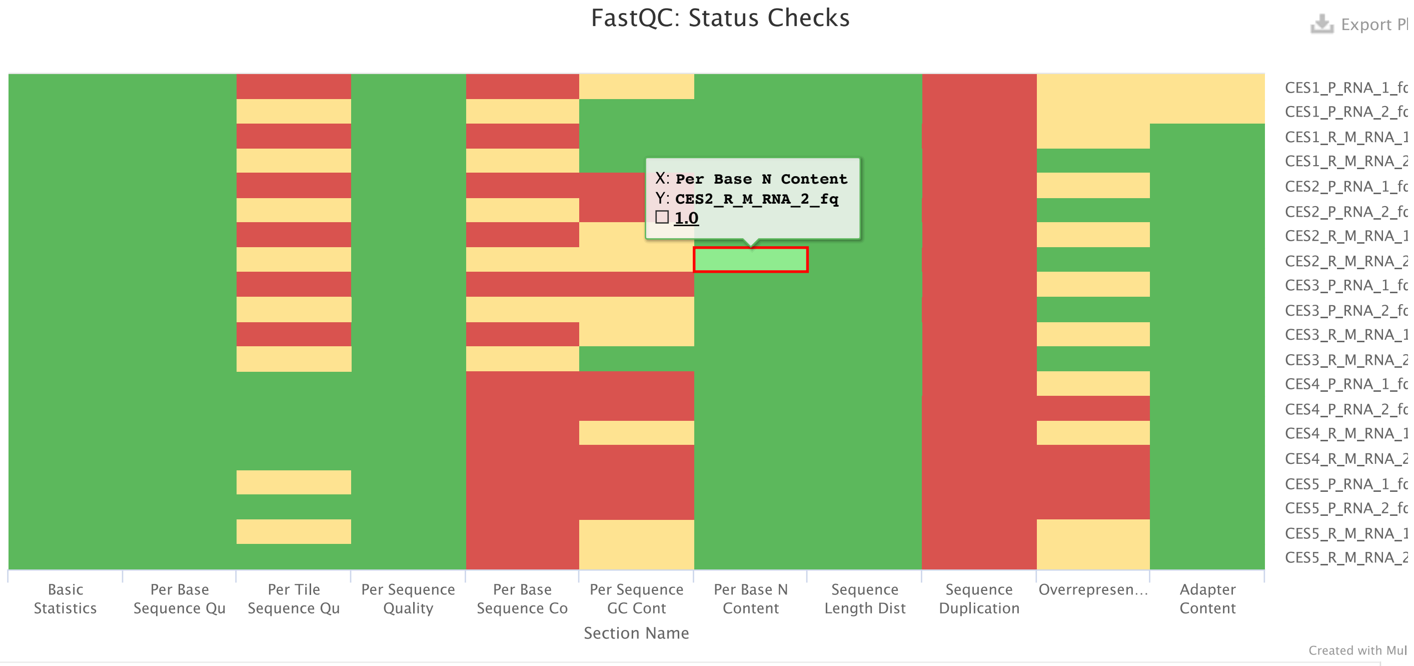

MultiQC v1.9 (http://multiqc.info) - Written by Phil Ewels (http://phil.ewels.co.uk), available on GitHub (https://github.com/ewels/MultiQC).
This report uses HighCharts (http://www.highcharts.com/), jQuery (https://jquery.com/), jQuery UI (https://jqueryui.com/), Bootstrap (http://getbootstrap.com/), FileSaver.js (https://github.com/eligrey/FileSaver.js) and clipboard.js (https://clipboardjs.com/).

SciLifeLab (http://www.scilifelab.se/)
