# Supplementary material for: Genomic, transcriptomic, and viral integration profiles associated with recurrent/metastatic progression in high‐risk human papillomavirus cervical carcinomas
Source: Cancer Med. 2020 Oct 5;9(21):8243–57. doi: 10.1002/cam4.3426 (PMC7643681; doi:10.1002/cam4.3426)
Supplement: Supplementary file 12 — Supplementary Material [file CAM4-9-8243-s012.pdf]

MultiQC

(http://multiqc.info)

A modular tool to aggregate results from bioinformatics analyses across many samples into a single report.

Report generated on 2020-06-15, 07:51 based on data in: `/Volumes/sequencing data/RNA seq2020/RNA BAM`

General Statistics

Copy table | Configure Columns | Plot | Showing 10/10 rows and 3/5 columns.

Sample Name	% Dups	% GC	M Seqs
CES1_P_FFPE_RNA_accepted_hits	70.8%	52%	58.0
CES1_R:M_FFPE_RNA_accepted_hits	63.2%	51%	79.3
CES2_P_FFPE_RNA_accepted_hits	63.7%	52%	92.7
CES2_R:M_FFPE_RNA_accepted_hits	50.2%	52%	125.7
CES3_P_FFPE_RNA_accepted_hits	63.3%	52%	96.5
CES3_R:M_FFPE_RNA_accepted_hits	54.0%	52%	117.0
CES4-P_FFPE_RNA_accepted_hits	97.3%	51%	88.5
CES4-R:M_FFPE_RNA_accepted_hits	98.2%	50%	84.2
CES5-P_FFPE_RNA_accepted_hits	99.1%	45%	146.6
CES5-R:M_FFPE_RNA_accepted_hits	76.7%	48%	113.5

FastQC

FastQC (http://www.bioinformatics.babraham.ac.uk/projects/fastqc/) is a quality control tool for high throughput sequence data, written by Simon Andrews at the Babraham Institute in Cambridge.

Sequence Counts

Help

Sequence counts for each sample. Duplicate read counts are an estimate only.

Number of reads | Percentages

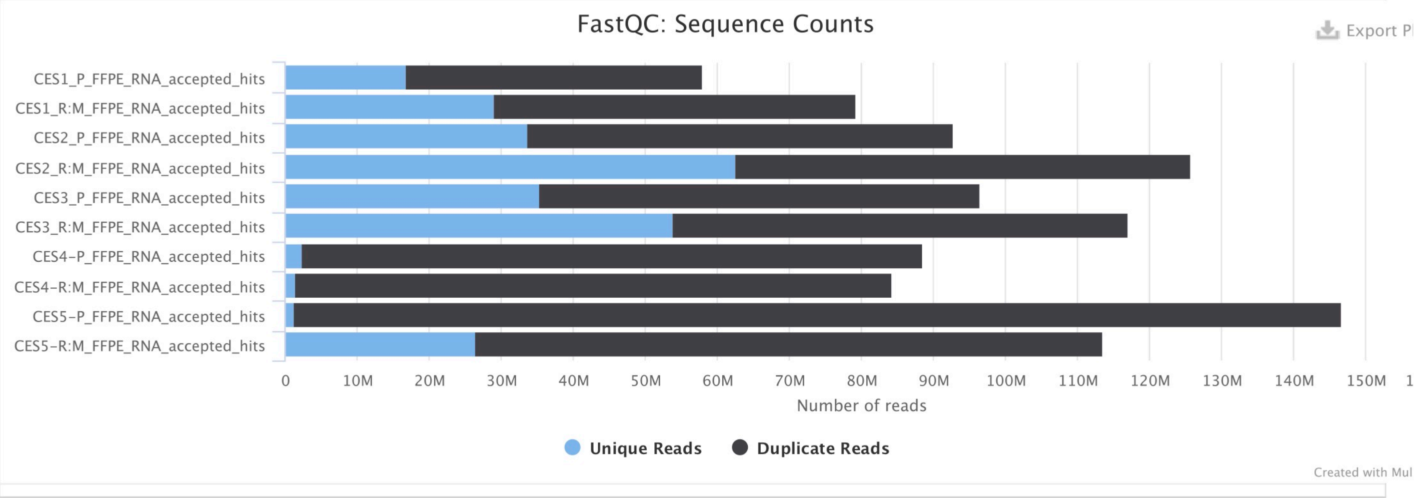

Sequence Quality Histograms 10

Help

The mean quality value across each base position in the read.

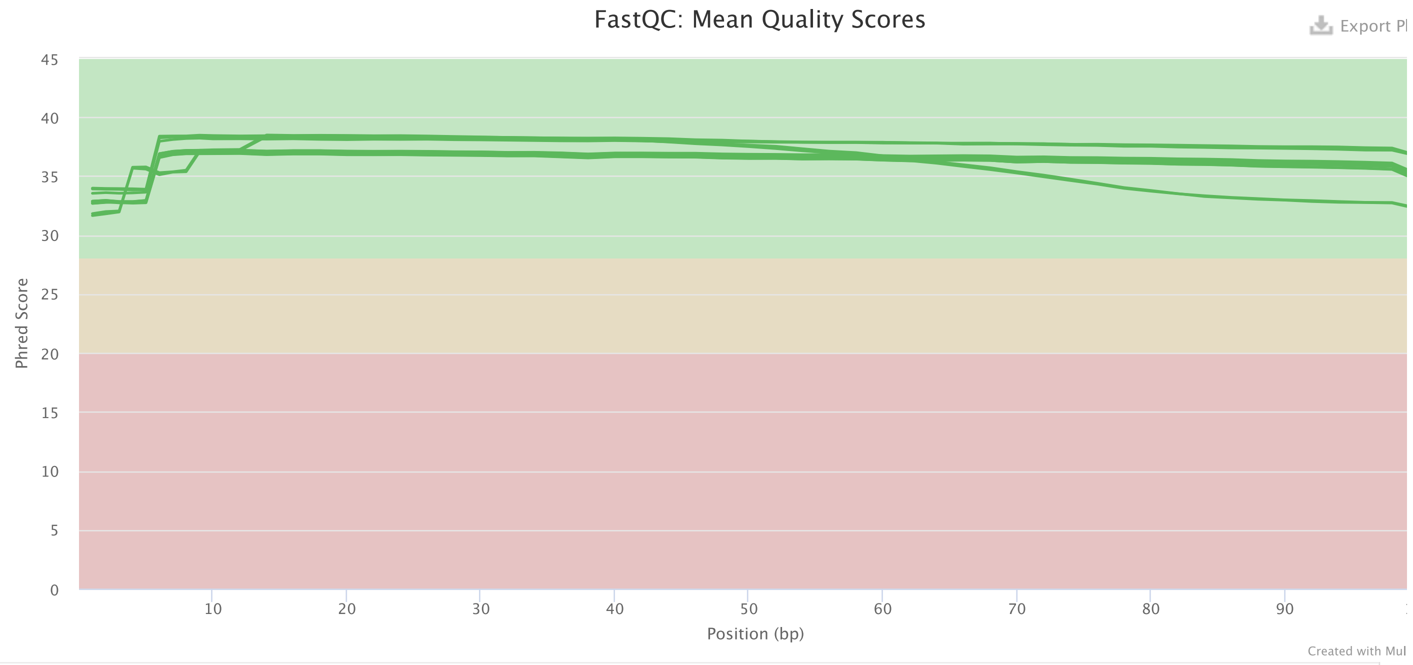

Per Sequence Quality Scores 10

Help

The number of reads with average quality scores. Shows if a subset of reads has poor quality.

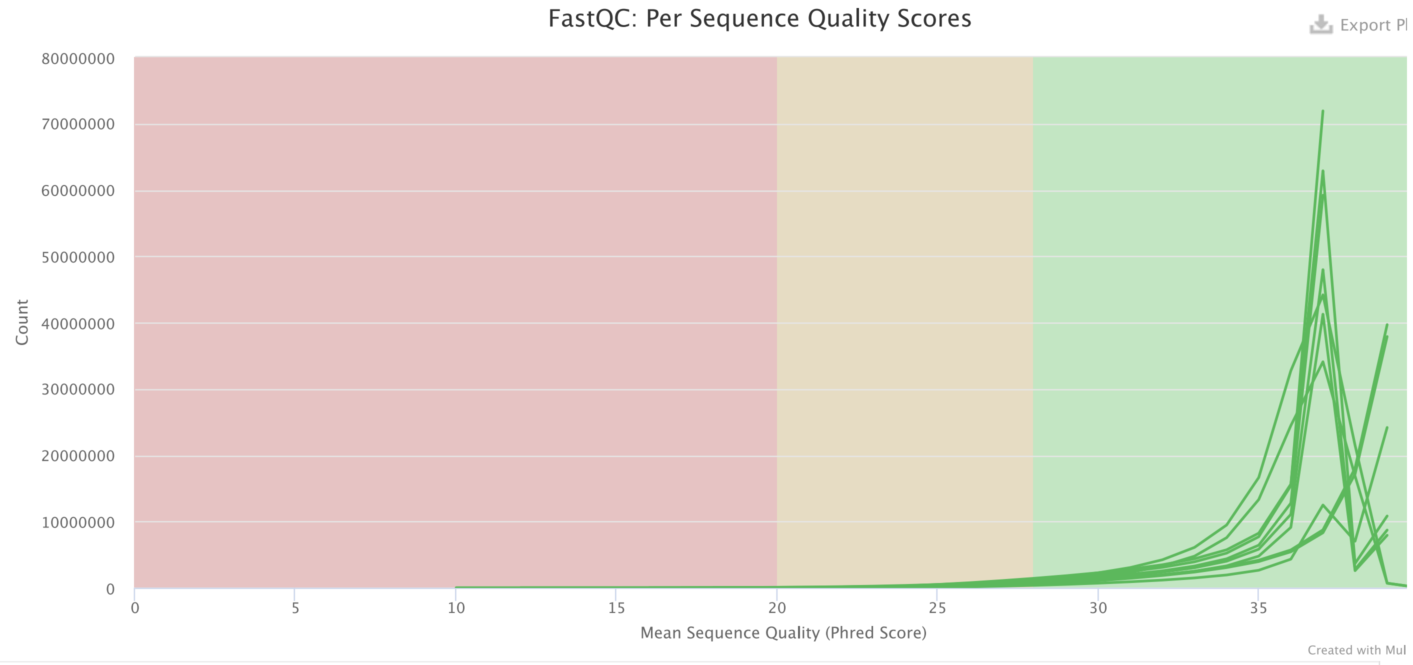

Per Base Sequence Content 02 8

Help

The proportion of each base position for which each of the four normal DNA bases has been called.

Click a sample row to see a line plot for that dataset.

Rollover for sample name

Position: - %T: - %C: - %A: - %G: -

Export Plot

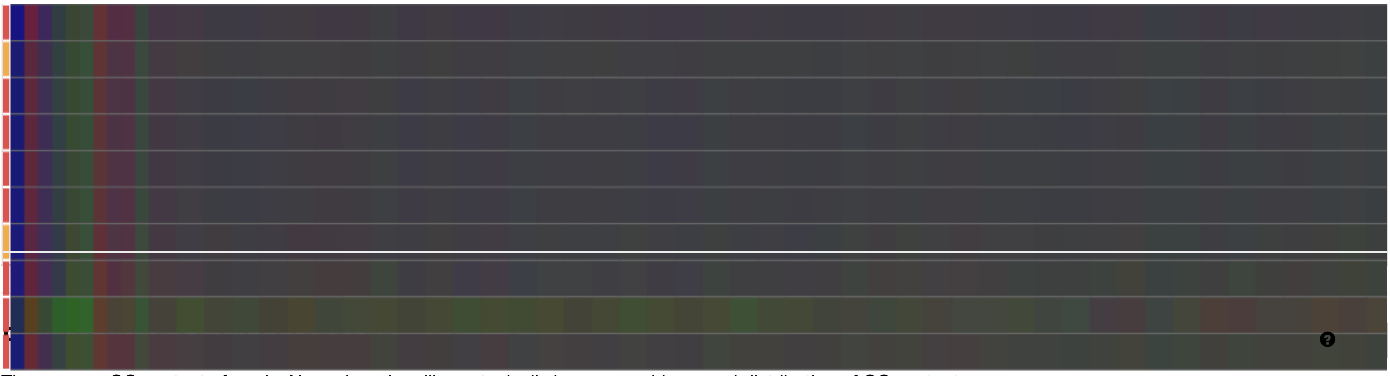

The average GC content of reads. Normal random library typically have a roughly normal distribution of GC content.

Percentages | Counts

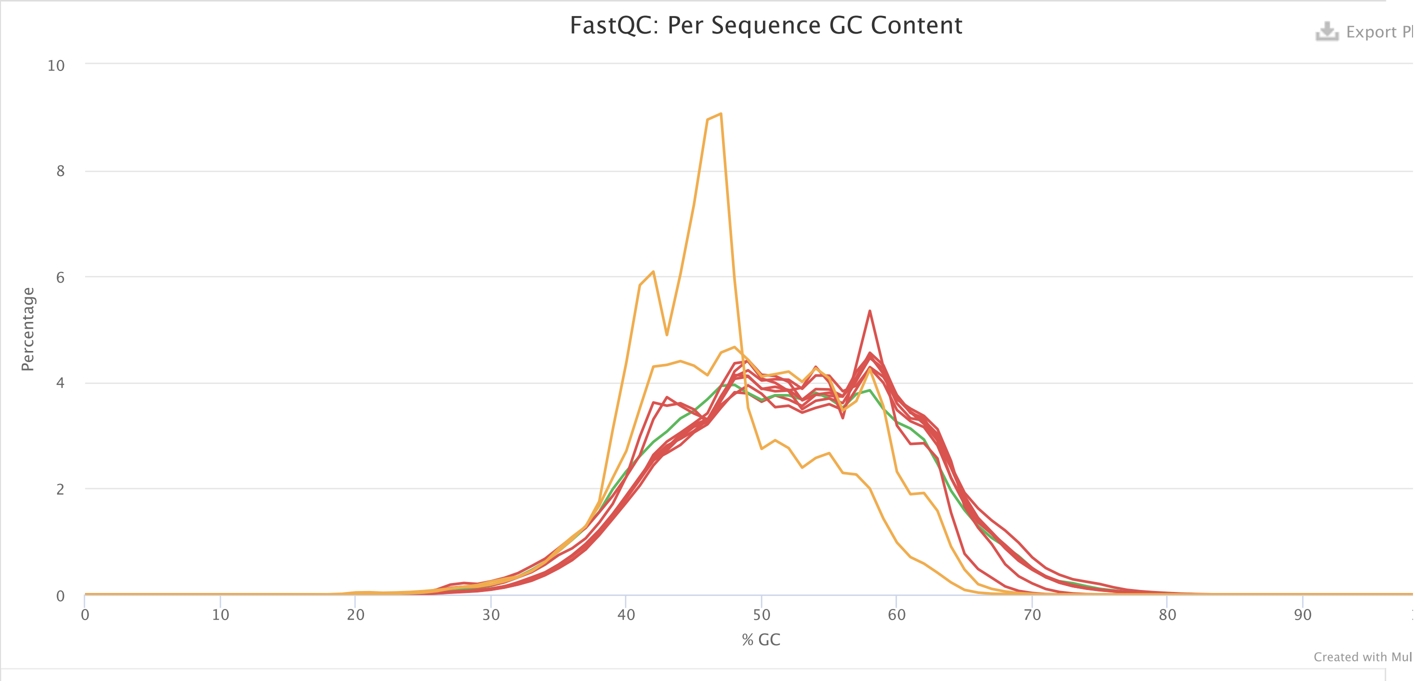

Per Base N Content 10

Help

The percentage of base calls at each position for which an `N` was called.

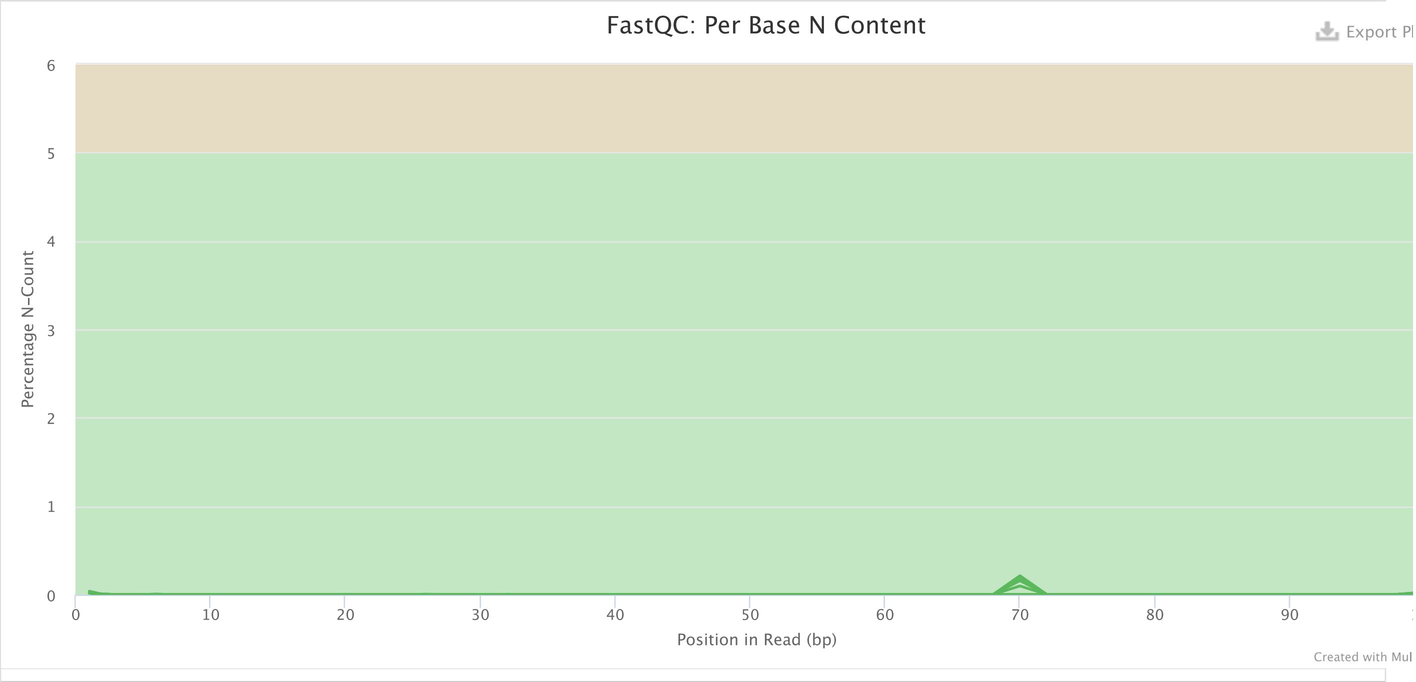

Sequence Length Distribution 10

All samples have sequences of a single length (101bp).

Sequence Duplication Levels 0 10

Help

The relative level of duplication found for every sequence.

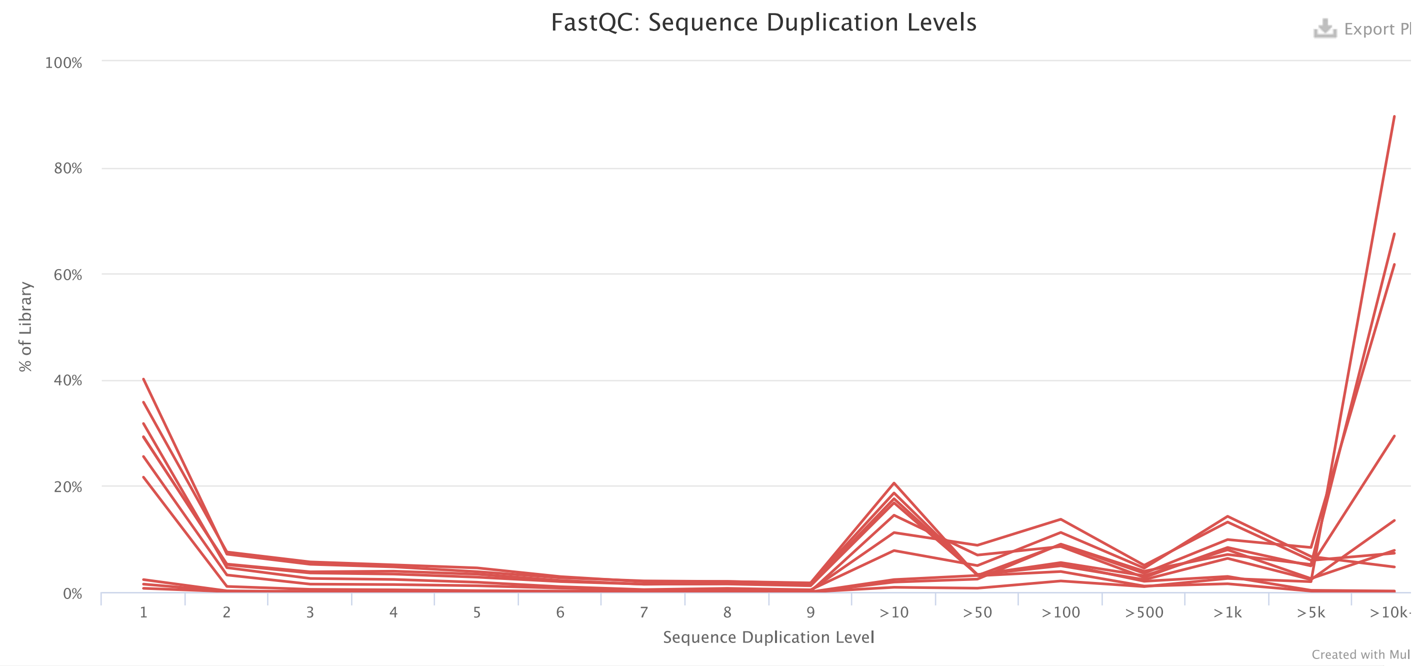

Overrepresented sequences 4 5 1

Help

The total amount of overrepresented sequences found in each library.

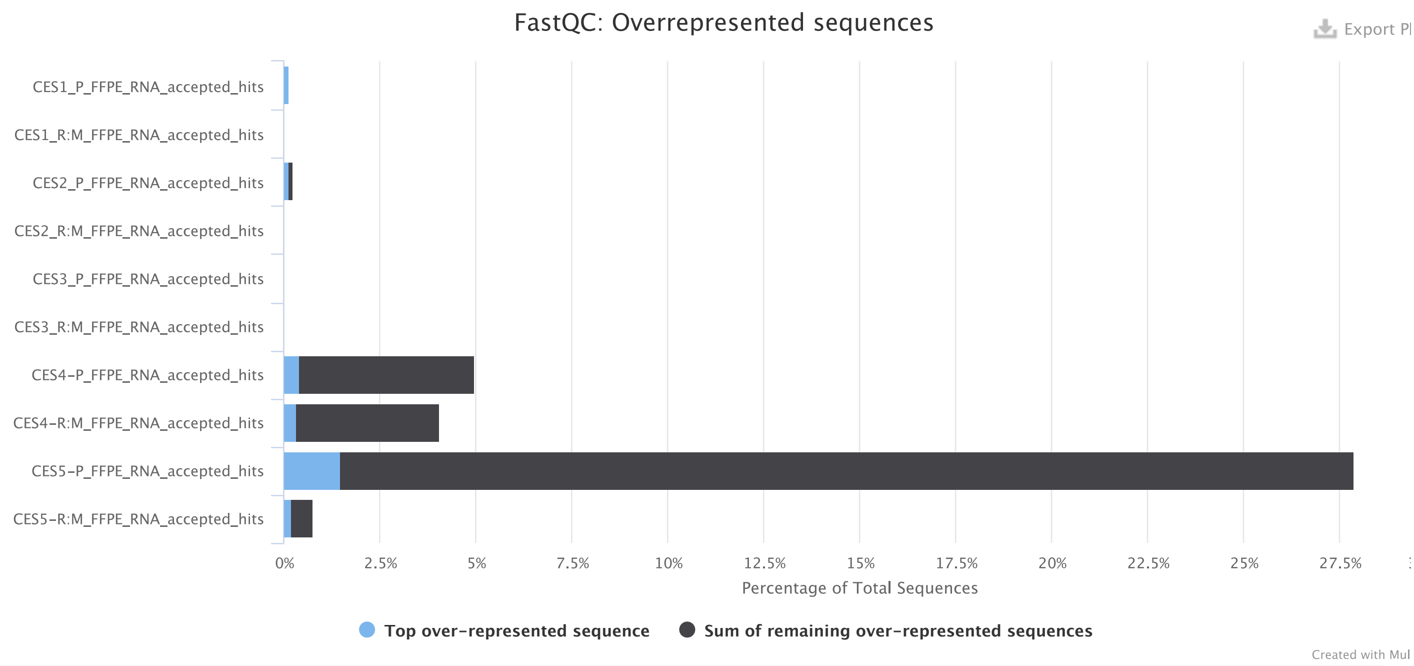

Adapter Content 10

Help

The cumulative percentage count of the proportion of your library which has seen each of the adapter sequences at each position.

No samples found with any adapter contamination > 0.1%

Status Checks

Help

Status for each FastQC section showing whether results seem entirely normal (green), slightly abnormal (orange) or very unusual (red).

Sort by highlight

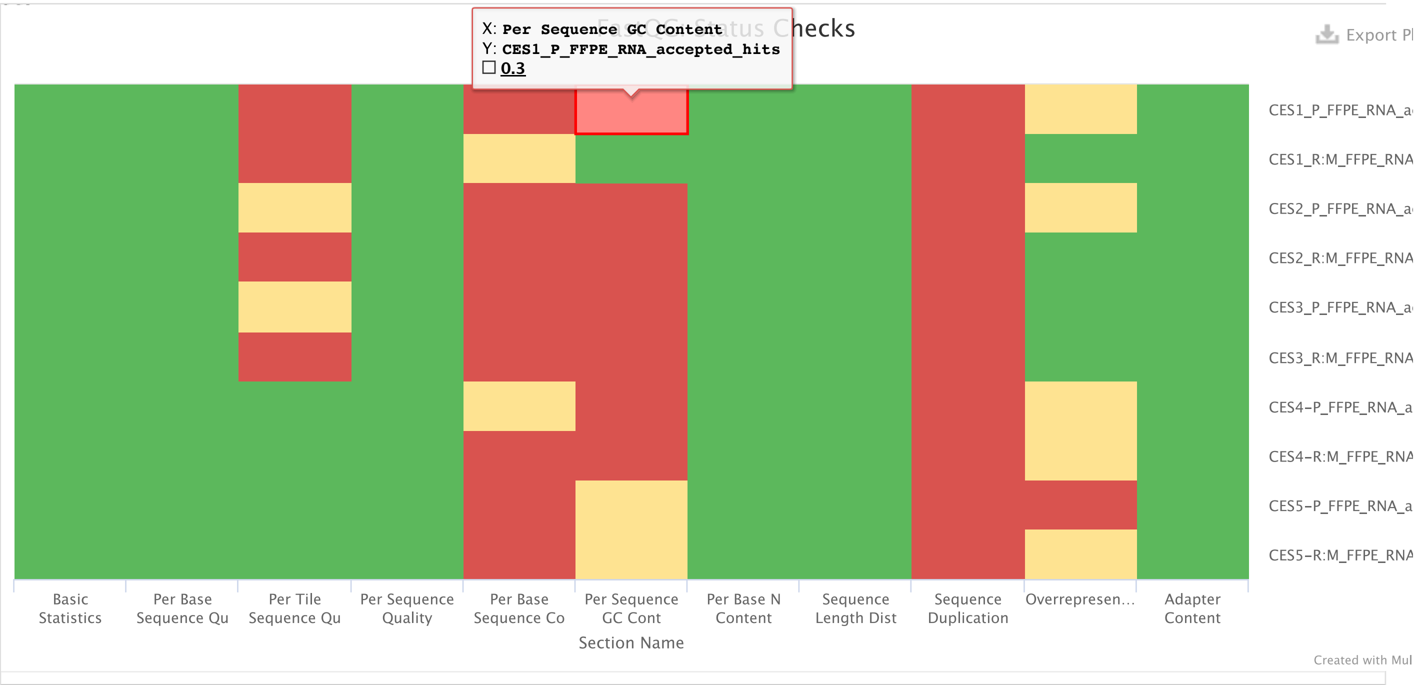

MultiQC v1.9 (http://multiqc.info) - Written by Phil Ewels (http://phil.ewels.co.uk), available on GitHub (https://github.com/ewels/MultiQC).
This report uses HighCharts (http://www.highcharts.com/), jQuery (https://jquery.com/), jQuery UI (https://jqueryui.com/), Bootstrap (http://getbootstrap.com/), FileSaver.js (https://github.com/eligrey/FileSaver.js) and clipboard.js (https://clipboardjs.com/).

SciLifeLab (http://www.scilifelab.se/)
