# Supplementary material for: Genomic, transcriptomic, and viral integration profiles associated with recurrent/metastatic progression in high‐risk human papillomavirus cervical carcinomas
Source: Cancer Med. 2020 Oct 5;9(21):8243–57. doi: 10.1002/cam4.3426 (PMC7643681; doi:10.1002/cam4.3426)
Supplement: Supplementary file 13 — Supplementary Material [file CAM4-9-8243-s013.pdf]

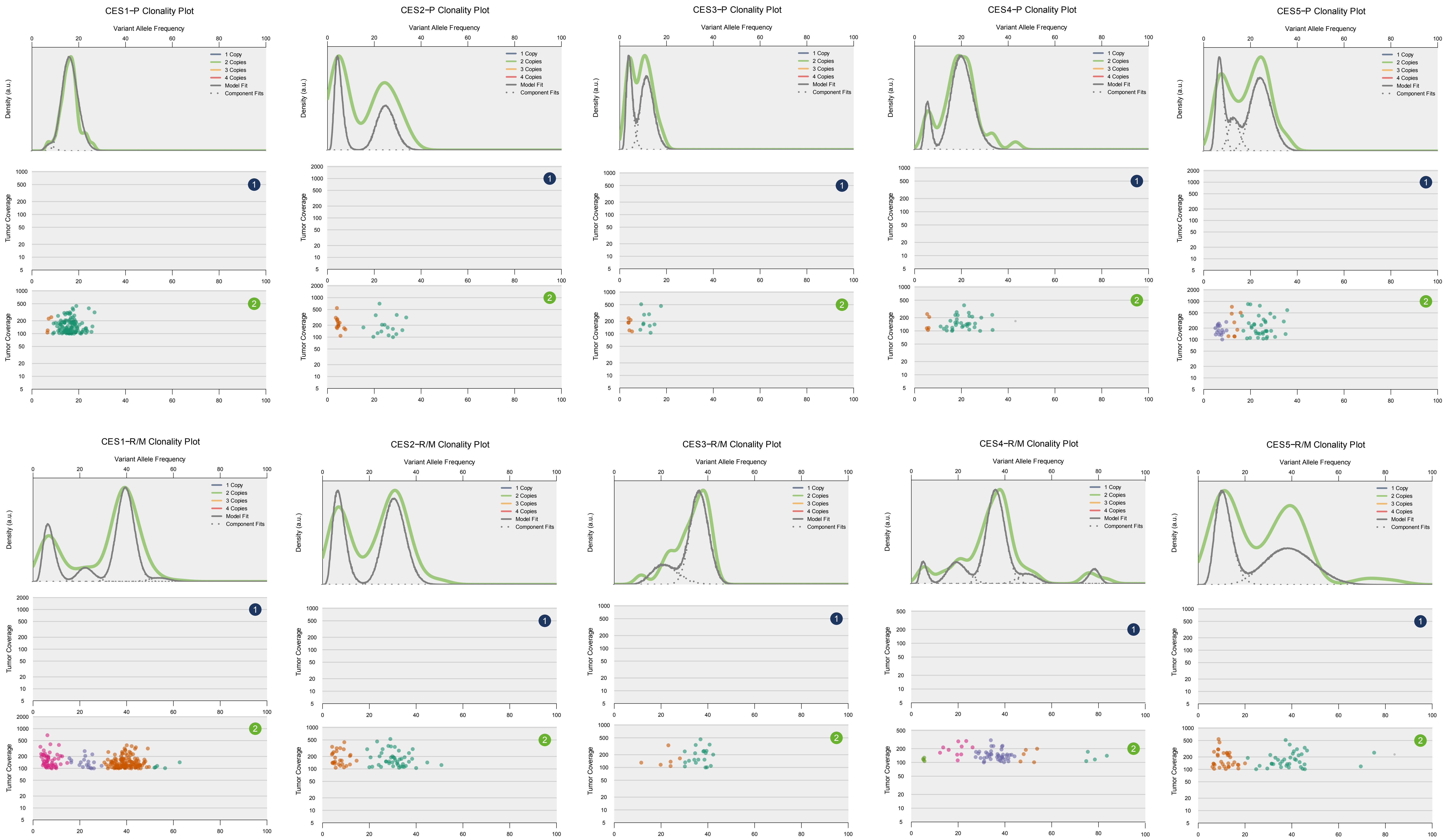
CES1−P Clonality Plot
CES2−P Clonality Plot
CES3−P Clonality Plot
CES4−P Clonality Plot
CES5−P Clonality Plot
CES1−R/M Clonality Plot
CES2−R/M Clonality Plot
CES3−R/M Clonality Plot
CES4−R/M Clonality Plot
CES5−R/M Clonality Plot
Variant Allele Frequency
Density (a.u.)
Tumor Coverage
1 Copy
2 Copies
3 Copies
4 Copies
Model Fit
Component Fits
1
2
